# Transitioning to Microplastic-Free Seed Coatings: Challenges and Solutions

**DOI:** 10.3390/polym16141969

**Published:** 2024-07-10

**Authors:** Rozenn Langlet, Romain Valentin, Marie Morard, Christine Delgado Raynaud

**Affiliations:** 1 Laboratoire de Chimie Agro-Industrielle (LCA), Univeristé de Toulouse, INRAE, Toulouse INP, 31030 Toulouse, France; rozenn.langlet@ensiacet.fr (R.L.); romain.valentin@ensiacet.fr (R.V.); 2 Bois Valor, OLMIX, 13 rue Jean Mermoz, 81160 Saint-Juéry, France; mmorard@boisvalor-olmix.com; 3 Centre d’Application et de Traitement des Agro-Ressources (CATAR), Toulouse INP, 31030 Toulouse, France

**Keywords:** microplastics, seed coating, biopolymers, regulation, REACH

## Abstract

This review addresses the issue of replacing manufactured microplastics in seed coatings used in agriculture. Firstly, it focuses on the policy and regulatory actions taken on microplastics at a global level. There is no consensus within the scientific community on the definition of a microplastic and, more generally, on the classification of plastic debris. Nevertheless, several decision schemes have been proposed in an attempt to define the notion of microplastics. The different criteria relevant to this definition, such as the size, physical state, chemical structure, origin, and persistence of microplastics, are discussed, with a comparison being made between the REACH regulation and the scientific literature. Seed production and processing are also discussed, with the functions of seed coatings being explained in order to gain a better understanding of the properties to be considered in a substitution strategy for currently used microplastics. The main challenges are multiple; substitutes must provide the same performance as microplastics: (i) improving the adherence of the treatment to the seed, (ii) distributing the treatment more evenly over the seed, (iii) reducing the amount of dust-off when handling treated seed, and (iv) improving the seed flowability, which is particularly important during the sowing stage, all while preserving the physiological properties of the seed. Substitute polymers are proposed according to the desired performance and functional properties: two main chemical families of biopolymers were identified in the literature: polysaccharides and proteins. Among them, 13 and 6 polymers, respectively, complied with REACH regulation, demonstrating adhesion, dust reduction performances, and preservation of seed physiological quality in particular. This work aims to guide future studies on microplastic substitution in seed coatings, and to highlight research needs in this area. It is based on an analysis and discussion of the literature, identifying and listing potential substitutes.

## 1. Introduction

The emergence of microplastics has resulted from the extensive production and use of plastics since the 1950s. Indeed, plastics have rapidly replaced a significant proportion of materials of natural origin, thanks to their low cost, ease of production, durability, and easily modifiable properties. However, the use of plastics also has major drawbacks. For instance, certain additives, such as specific flame retardants, perfluorinated products, phthalates, bisphenols, and nonylphenols, are toxic [1]. Moreover, some plastics exhibit poor degradability and difficulty in recycling [2], leading to laborious end-of-life management.

The most widely used polymers are polypropylene (PP), low-density polyethylene (LDPE), polyvinyl chloride (PVC), high-density polyethylene (HDPE), polyethylene terephthalate (PET), polyurethane (PUR) and polystyrene (PS), collectively accounting for approximately 75% of global plastic production (390.7 Mt) in 2021 [3]. These polymers also account for 92% of all plastics ever produced (7800 Mt) [4]. As a result, they are the most common plastics found in the environment, particularly in aquatic environments [5]. According to an IUCN brief from 2021 [6], at least 14 million tons of plastics are discharged into the oceans annually. These releases into the environment have been shown to cause significant damage to flora and fauna [7,8].

Once in the environment, plastic can take up to several thousand years to be completely degraded [9]. During this process, the plastic becomes brittle, and then fragments before starting to degrade under various environmental factors. However, there are also microplastics manufactured for industrial use (pellets) or domestic use (such as polymers used to encapsulate certain active ingredients).

The first scientific observation of a plastic fragment in the environment was made in 1972 [10] in the Sargasso Sea, where particles were found to be mainly between 0.25 and 0.5 cm in diameter. It was not until 1990 that the term “microplastic” was first used [11], defined as plastic debris with a diameter of less than 20 mm. Over time, the upper and lower size limits have been progressively reduced, leading to the coexistence of numerous definitions in the scientific literature. Only recently have consensus proposals emerged [12,13].

Estimating the global quantity of microplastics is challenging due to their wide geographical presence: oceans (surface, sediments) [14], rivers and lakes [15], groundwater [16,17], beaches [18], soils [19], snow [20,21], fauna [22], flora [23], and humans [24]. Furthermore, the geographical distribution of microplastics is influenced by a number of factors, including their density, currents, and winds [25]. Finally, the development of standardized methods for collecting and analyzing microplastics has only recently become available [26,27,28], and the data needed for a completer and more reliable estimate of microplastics are still being acquired.

In 2019, Eriksen et al. [29] estimated the average annual number of particles floating on the surface of the oceans to be between 82 and 358 thousand billion, or between 1.1 and 4.9 million tons. The study also shows a rapid and significant increase in these figures since 2005, a trend also observed for particles collected on beaches worldwide. Isobe et al. [30] estimated this number to be 24.4 trillion particles for the same year. Therefore, microplastics are ubiquitous and in significant quantities.

It is also crucial to assess the impact of these materials on human health and the environment. Microplastics present a serious concern due to their long-lasting presence in the environment, combined with their small size, their capacity to transport toxic elements, and their high concentration in additives.

The incorporation of additives into plastics aims to enhance their properties and extend their lifespan, yet this practice also accentuates their persistence while having additional harmful effects on the environment [31].

The small size of microplastics facilitates their transportation by air or water. Consequently, they can contaminate the entire food chain and ultimately reach our plates [32]. This size also allows other routes of exposure, such as inhalation and penetration through the skin barrier. Because of this characteristic, combined with their persistence, some of the microplastics that have penetrated organisms can accumulate in the human body [33]. The term “nanoplastics” has recently been coined to describe even smaller plastic fractions. Their presence has been observed in soils [34] and oceans [35].

Finally, microplastics are capable of adsorbing heavy metals [36] and organic pollutants [37] thanks to their high specific surface area. The adsorption capacities for each heavy metal depend on the chemical structure of the microplastic [36]. Furthermore, it has been demonstrated that microplastics with high hydrophobicity have a high adsorption capacity for hydrophobic organic pollutants [37].

These characteristics, when considered alone and/or in combination, have the potential to cause adverse effects on human health. A review by Li et al. [38] provides a summary of these effects, which include oxidative stress, DNA damage, organ dysfunction, metabolic diseases, immune response, and neurotoxicity. However, it should be noted that these results are based on experimental models, and there is a lack of knowledge regarding the actual effects on the human body. Nevertheless, some epidemiological results described in the review suggest that several chronic diseases in humans could be linked to exposure to microplastics. It remains unclear whether the observed adverse effects of microplastics on human health are generalized to all types of microplastic or specific to a particular type. In order to address microplastic pollution, a number of initiatives and regulations have been implemented worldwide.

This review focuses in particular on the issue of replacing manufactured microplastics contained in seed coatings used in agriculture, which constitutes an often-overlooked source of soil and air contamination. The various actions taken on microplastics, whether from a political or regulatory point of view, are discussed. Families of substitute polymers are proposed based on the actual European regulation, performance targets, and functional properties required for seed-coating agents, as expressed in this article. The final and innovative objective of this work is to guide future studies on the substitution of microplastics in seed coatings by proposing solutions and highlighting research needs on the subject.

## 2. State of the Art on Current Initiatives and Regulations against Microplastics

**Figure 1:**
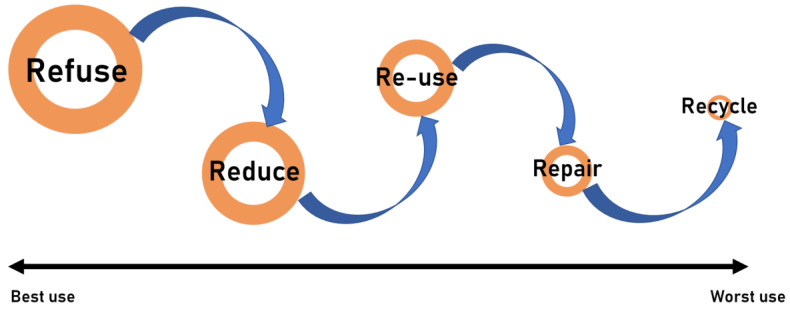
The 5Rs principle for the prevention of plastic pollution, adapted from [39].

A number of strategies can be employed to combat the generation and/or release of microplastics. The relative impact of these strategies can be gauged by reference to the 5Rs principle, as illustrated in Figure 1.

### 2.1. Actions to Restrict the Discharge of Plastic Waste

Given the scale of the microplastic problem, the complete removal of microplastics from the environment is unfeasible. Therefore, the most effective solutions are those that eliminate microplastics at the source. According to Boucher and Friot [40], between 4.8 and 12.7 Mt of microplastics resulting from the fragmentation of plastic waste are added to the oceans every year.

#### 2.1.1. Restrict the Use and Production of Plastic Objects

**Figure 2:**
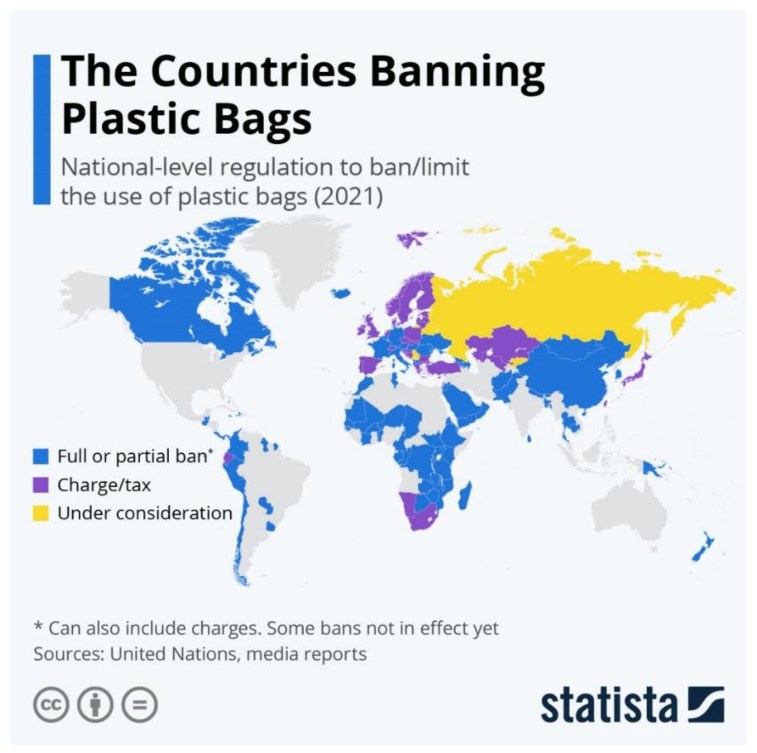
Planisphere depicting countries that have enacted bans or restrictions on the use of single-use plastic bags [43].

Prior to the discovery of microplastics, numerous countries had already initiated measures to reduce plastic pollution. One of the most notable measures aims for the reduction in the use of single-use plastic bags made from LDPE. In 2002, Bangladesh became the first country to ban the production of single-use plastic bags [41]. Figure 2 illustrates the countries that enacted bans or restrictions on single-use plastic bags by 2021. Australia joined this list in 2022 with the introduction of a ban on the sale of plastic bags in 2021 [42].

Between 2018 and 2021, the consumption of lightweight plastic bags (bags with a thickness of between 50 and 15 µm) fell by 31% in the European Union as a result of the implementation of the “The Plastic Bags Directive” [44] in 2015 (there were no consumption measurements before 2018). In 2018, 127 of the 192 countries (66%) had enacted regulations to manage plastic bags [45].

Another lever for action is the banning or limiting of single-use plastic objects. First measures have been implemented at the local level, such as the first ban on the use of single-use coffee pods in public institutions in 2016 in the city of Hamburg, Germany [46]. In 2017, Zimbabwe banned the use of expanded polystyrene, used for food containers [47]. In 2018, Vanuatu enacted legislation banning the use of polystyrene food containers and single-use plastic straws [48]. In 2020, France implemented a ban on the purchase of plastic plates, cups, straws, cotton buds, and other single-use objects [49]. In 2022, it imposed a ban on plastic packaging for the sale of certain fruits and vegetables [50]. In 2023, the country became the first to prohibit the use of disposable tableware in fast-food restaurants for meals consumed on the premises [49].

On a larger scale, the European Union has enacted a ban on the use of plastic cotton buds, balloon sticks, and single-use plastic takeaway containers and catering items, effective in 2021 [51].

At the global level, the United Nations General Assembly adopted a resolution in March 2022 [52] to establish a “global treaty against plastic pollution” by the end of 2024. Negotiations on this text involve 193 countries over a two-year period. The objective is to achieve a binding text with measures that encompass the entire life cycle of plastics, from production to end of life. This text also aims to “put an end to plastic pollution by 2040”.

Finally, if the plastic object has already been manufactured, it is necessary to encourage the implementation of circular economic models that promote reuse and repair rather than disposal.

#### 2.1.2. Restrict the Discharge into the Environment

In instances where the disposal of plastic waste is unavoidable, there are a number of potential management routes for the waste: recycling, recovery of the waste into energy or resources, landfill, or direct release into the environment.

According to the OECD [53], in 2019, 49% of global plastic waste was landfilled, 22% was poorly managed or collected and discharged directly into the environment, 19% was incinerated for energy recovery, and only 9% was recycled.

According to PlasticEurope [3], in the European Union in 2020, the proportion of plastic waste recycled was 35% on average, with 42% of waste transformed into energy, and 23% landfilled. In France, only 25% was recycled, and 31% landfilled. The same organization makes no mention of the waste eventually released into the environment.

There are several ways to increase the proportion of recycled plastic waste. These include the following:

1. Using recyclable plastic materials (such as PET, PE, or PP), simplifying the polymer blends used, and reducing the use of materials that are difficult to recycle, from the design stage of plastic products onwards.
2. Raising public awareness of the importance of recycling, the associated benefits, and good sorting practices.
3. Setting up selective waste collection infrastructures, and improving the efficiency of existing infrastructures.
4. Expanding the range of recycled plastics as much as possible.
5. Investing in the research and development of new recycling technologies.

The Eionet ETC report [54] indicates that between 2012 and 2018, the quantity of waste from plastic packaging and small unpackaged plastic objects (PPSIs) increased in the majority of European countries, regardless of whether it was properly managed or not. Consequently, the collective efforts made during this period were not sufficient to reduce plastic discharges into the environment within the European Economic Area. Six countries did, however, manage to reduce the amount of poorly managed plastic waste between 2012 and 2018: Finland, Ireland, Lithuania, Poland, Slovenia and Croatia. These results are mainly due to the expansion of collection areas, programs to combat illegal landfills, and improved landfill management. It is of utmost importance to continue raising public awareness of the necessity to refrain from disposing of plastic waste into the environment.

These various operations can serve as exemplars for other countries to reduce the amount of waste discharged into the environment on a global scale.

### 2.2. Actions to Limit the Release of Manufactured Microplastics

In addition to the fragmentation of larger plastic objects, microplastics also exist in products where they are deliberately used in this form, i.e., manufactured microplastics. An IUCN report [40] from 2017 estimates the quantity of these microplastics discharged into the oceans at 16,000 to 50,000 tonnes per year, considering only the microplastics included in hygiene products. The European Chemical Agency (ECHA) [55] estimates annual discharges in the European Union at 42,000 tonnes (between 13,000 and 95,000 tonnes), and considers several categories of products: cosmetics, but also detergents and cleaning products, products for agricultural and horticultural use, oil and gas industry products, paints and coatings, in vitro diagnostic products, medicinal products, food additives, and synthetic sports surface materials.

To address this issue, various measures have been implemented globally.

#### 2.2.1. Plastic Microbeads

Initially, global attention was focused on the plastic microbeads present in cosmetic products, as these are microplastics whose source is relatively straightforward to identify and regulate.

As scientific knowledge about microplastic pollution has advanced, several non-profit organizations have taken action including 5 Gyres, Plastic Soup Foundation, and Greenpeace. These actions have led to reactions from industry and government institutions.

The most notable awareness campaign was launched by the Plastic Soup Foundation in 2012, “Beat the Microbead” [56], which raised awareness not only about microbeads but also about microplastics in general. This campaign even led to the creation of a mobile application enabling users to check cosmetic products for the presence of microplastics by scanning them directly with their phones.

In 2013, 5 Gyres initiated the Beat the Bead action campaign [57], which led to the prohibition of plastic microbeads in rinse-off cosmetics in several US states. This was followed by a nationwide ban in 2015 with the enactment of the Microbead-Free Waters Act [58]. This legislation prohibits the manufacture, packaging, and distribution of rinse-off cosmetics and over-the-counter medicines, including toothpastes containing plastic microbeads.

Plastic microbeads are defined as “any solid plastic particle that has (i) a size less than or equal to 5 mm; (ii) been manufactured for the purpose of exfoliating or cleansing the whole body or any part of the body”.

The law (i) excludes any biodegradable alternative and (ii) does not take into account other leave-on cosmetic products (make-up, deodorant, lotions, etc.) [59]. However, this is the first nationwide measure to combat a proportion of manufactured microplastics.

In 2017, Canada followed the U.S. model with the “Microbeads in Toiletries Regulations” [60], published in 2017. This legislation is analogous to the “Microbead-Free Waters Act” concerning the measures taken, the definition of microbeads, and the products concerned.

In 2018, 8 of the 192 countries (4%) established bans on microbeads through national laws or regulations: the United States, the United Kingdom, Sweden, New Zealand, Italy, South Korea, Canada, and France. Ireland subsequently passed a similar law in 2019 [61].

In 2017, Belgium entered into a bipartite agreement between the Ministry of Energy, the Environment and Sustainable Development, and the association of producers and distributors of cosmetics and detergents (Detic). The objective of this agreement was to eliminate microplastics from certain finished products with a particular focus on primarily rinse-off cosmetics and toothpaste [62].

**Figure 3:**
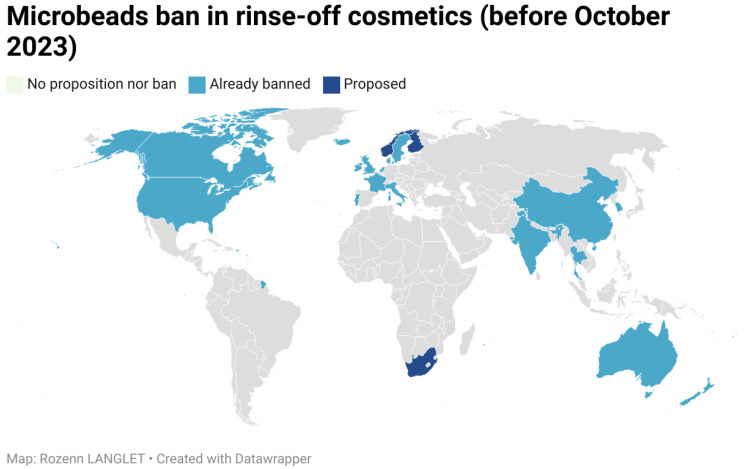
Planisphere listing countries that have enacted bans or are in the process of enacting legislation to prohibit the use of microbeads in rinse-off cosmetics, completed from [63,64].

In 2019, the Plastic Soup Foundation created a map which listed the countries that had banned or were considering banning microbeads in rinse-off cosmetics (Figure 3).

#### 2.2.2. Manufactured Microplastics

In light of the current state of knowledge regarding microplastics, the European Commission requested in 2017 that the European Chemicals Agency (ECHA) prepare an application dossier for REACH restrictions on the marketing of products intentionally containing microplastics. The final text (regulation 2023.2055) was finally adopted on 25 September 2023 and applied from 17 October 2023 [65].

This restriction only applies to microplastics intentionally used in manufactured products. The regulation prohibits the placing on the market of synthetic polymer microparticles as a substance (chemical element and its compounds in their natural state or obtained by a manufacturing process) or in mixtures (mixture or solution composed of two or more substances) at concentrations equal to or greater than 0.01% by mass.

**Figure 4:**
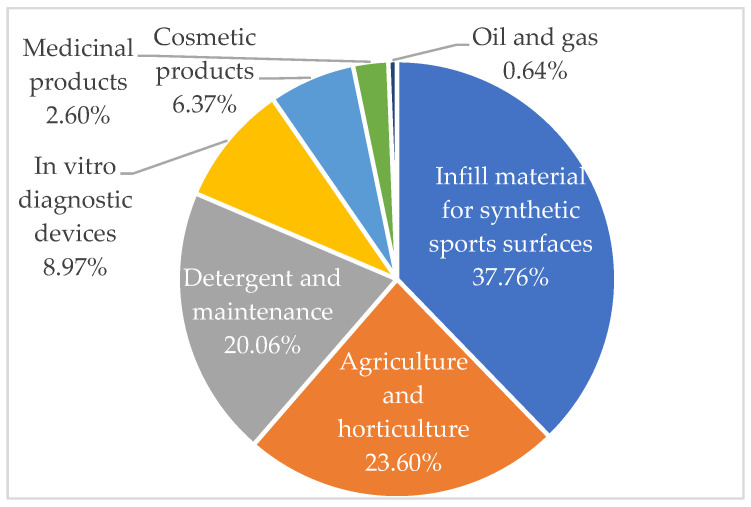
Distribution of intentionally added microplastic discharges per year by sector, as reported by ECHA.

A diverse array of sectors is implicated, including cosmetics, detergents and maintenance products, agricultural and horticultural products, oil and gas, paints and coatings, construction products, in vitro diagnostics, medical devices, medicines, food additives, and synthetic sports surface materials. The respective tonnages released into the environment for each field are depicted in Figure 4.

The text indicated that this regulation should result in a reduction in manufactured microplastics discharged into the environment by 500,000 tons over a 20-year period.

This is the most ambitious regulation to date concerning microplastics, in terms of the scope of the restrictions and the countries involved. No measures involving all these areas have been taken in other interstate unions.

In this review, we focus on manufactured microplastics in seed coatings used in agriculture. To understand this issue, we first need to define microplastics.

## 3. Definitions of Microplastics (MPs) and Their Differences

After the first use of the word “microplastic” by Peter G. Ryan [11] in 1990, and its democratization by Thompson et al. [66] in 2004, the word was initially used to describe “plastic fragments smaller than 1 mm in size” [67,68].

However, the definition of a microplastic and the overall classification of plastic debris lack consensus within the scientific community [69]. Despite this, several decision schemes [69,70] have been proposed in an attempt to define the notion of microplastic in the scientific literature.

The various criteria impacting this definition, such as the size, physical state, chemical structure, origin, and persistence of microplastics, are subsequently discussed on the basis of the scientific literature and REACH regulations.

### 3.1. Comparison of Definitions: REACH versus Literature for Each Criterion

In 2023, European Regulation 2023.2055 amending REACH Annex XVII (or Restriction List) proposes its own definition of a microplastic: “synthetic polymer microparticles”, i.e., polymers in a solid state, fulfilling the following conditions:

1. Either contained in particles and constituting at least 1% by weight of said particles, or building a continuous surface coating on said particles.
2. At least 1% by weight of the particles described in 1. must have (i) all dimensions between 5 mm and 0.1 µm; (ii) in the case of fibers, a length less than or equal to 15 mm, any dimension greater than or equal to 0.3 µm, and a length/diameter ratio > 3.

The following polymers are exempt from this definition:

- Polymers which are the result of a polymerization mechanism that already occurs in nature, and which are not chemically modified substances (within the meaning of the REACH regulation).
- Polymers that are biodegradable as proven by certain criteria detailed further.
- Polymers with a water solubility greater than 2 g/L as proven by certain methods detailed further.
- Polymers containing no carbon atoms in their chemical structure.

#### 3.1.1. Size

Size is the most often used criterion to categorize different types of plastic debris. This can be explained by the fact that it is a criterion of great importance for understanding their interaction with flora and fauna [71] and determining their fate in the environment.

According to the Literature

In 2008, the U.S. National Oceanic and Atmospheric Administration (NOAA) organized the first research workshop on the subject: a consensus was reached, stipulating that microplastics would be defined as plastic particles smaller than 5 mm in size [72]. This decision was justified on the one hand for practical reasons: it corresponds to the upper size limit of the Neuston nets normally used to capture plankton and floating debris, and on the other hand to not consider the physical obstruction of the gastrointestinal tract when analyzing the potential harmful effects of plastic debris on the environment.

In 2019, Frias and Nash [12] proposed a definition maintaining this limit, while adding a lower size limit of 1 µm, in order to advance the discussion on a general definition within the scientific community.

However, the 5 mm limit is debated: in 2023, Chae et al. [73] suggested lowering the limit to 1 mm, considering several criteria: (i) the nomenclature of the international system corresponding to the prefixes mini and milli, (ii) the particle size distribution of the majority of MPs found in the environment, which mostly corresponds to MPs smaller than 1 mm, and (iii) the fact that existing studies on the biotoxicity of MPs focus on sizes smaller than those recommended, in order to correspond to MP sizes that organisms studied could ingest.

In 2020, the International Organization for Standardization (ISO) suggested a new nomenclature for the international system, in which microplastics could be classified into 2 groups: a first group with dimensions between 1 mm and 5 mm (large microplastics) and a second group with dimensions between 1 µm and 1 mm (microplastics) [74].

Despite this effort, the upper limit of 5 mm is still widely adopted in recent studies [75,76,77,78,79] without mentioning the ISO size distinction.

The lower size limit is either not mentioned [75,76,77,78,79] or varies greatly from one study to another [13,73]. The size categories studied are often determined on the basis of the sampling method, or the technical observation limitations. The NOAA workshop [72] proposed a lower limit of 333 µm, which corresponds to the size limit of Neuston nets. However, according to Hartmann et al. [69], this definition should not be based on current methodological and analytical capabilities, since these are constantly evolving.

On the other hand, Bermùdez and Swarzenski [13] proposed that the definition of microplastics should include particles at least 2 µm in size, based on the size range potentially ingested by zooplankton.

The first appearance and definition of the term “nanoplastic” [80] in 2012 makes it possible to propose a lower limit, thus framing the scope of the term “microplastic”. According to this definition, which is based on the European Commission’s recommendation published in 2011 [81] concerning the definition of manufactured nanomaterials, nanoplastics are “plastic particles smaller than 0.1 µm”. Similarly, The International Union of Pure and Applied Chemistry (IUPAC) defines nanoparticles as “particles of any shape with dimensions between 1 nm and 100 nm” [82].

Gigault et al. [83] point out that such a definition is inappropriate, as it can lead to confusion between nanoplastics and engineered nanoparticles, which they consider to be different due to their different production routes and physico-chemical properties. They propose their own definition: nanoplastics are “particles within a size ranging from 1 to 1000 nm, resulting from the degradation of industrial plastic objects and which can exhibit colloidal behavior”. The upper size limit of 1 µm is currently the most widely used to qualify nanoplastics [84,85,86]. However, the distinction between nanoplastics and engineered nanoparticles (such as latex-like polymer nanoparticle dispersions) is still rarely made in the literature, since engineered polymer nanoparticles (PS, PE) are the most widely used experimental model for studying nanoplastics [85,86,87,88].

According to REACH

REACH’s upper limit of 5 mm is in line with the majority of definitions found in the literature. The 0.1 µm limit, on the other hand, comes from the European recommendation concerning the definition of manufactured nanoparticles [81].

Contrary to the majority of articles found in the literature, the REACH regulation adds the case of microfibers to its definition, with specific dimensions: length less than 15 mm, all dimensions greater than 0.3 µm, with a length/diameter ratio > 3.

Indeed, according to the European text, “some synthetic fibers are longer than 5 mm, but shorter than 15 mm, such as particles used to reinforce adhesives and concrete”.

Microplastics from fibers used for concrete reinforcement are also reported in the literature [89]. However, the dimensions of these fibers are not taken into account in the definition of microplastics. The size criteria for microfibers are in most cases similar to those for microplastics: their dimensions must be between 5 mm and 1 µm. They are even referred to as “microplastic fibers” [90]. The most common definition is “any natural or artificial fibrous material with a filamentary structure whose diameter is less than 50 µm, whose length is between 1 µm and 5 mm, and whose length/diameter ratio is greater than 100” [91]. The length/diameter ratio criterion considered is therefore also different.

In 2020, Suaria et al. [92] studied microfibers found in ocean surface waters: out of 23,593 fibers collected, they showed that less than 0.5% were longer than 5 mm and had a diameter greater than 30 µm. Only 3 fibers out of 23,593 were longer than 15 mm. The most common length category was between 0.8 and 0.9 mm, with a diameter between 15 and 17 µm. Very few fibers were less than 10 µm in diameter (less than 0.5%). The length/diameter ratio was not specified. Thus, few fibers larger than 5 mm and smaller than 10 µm in diameter were found in ocean surface waters.

#### 3.1.2. Physical State

In the majority of studies, the solid nature of microplastics is implied.

In practice, most polymers belong to the solid class according to the criteria of the Globally Harmonized System of Classification and Labelling of Chemicals (GHS). However, some polymers do not have a Tf, but a glass transition temperature (Tg) above which they become progressively viscous and fluid, without actually “melting”. In some cases, (i) this Tg is lower than room temperature (T_amb_) (such as certain amorphous polymers), or (ii) the polymers have a Tg (<T_amb_) and a Tf, and between these two temperatures, they will be viscous and relatively fluid (such as certain semi-crystalline thermoplastic polymers). These temperatures vary greatly according to the structures of the polymers, the added additives, and possibly their state of degradation.

According to the Literature

For the case where polymers have a Tg (<T_amb_) and a Tf, Verschoor [70] considers the melting temperature criterion to be the best practical option, since this information is usually given on the safety data sheet (SDS), which does not require additional testing. This option amounts to following the GHS criteria without taking into account the presence of a glass transition. The author suggests, however, that scientific discussion should focus on the possible adverse effects of these types of polymers on flora and fauna in order to really understand whether they should be excluded from the definition or not.

On the other hand, Hartmann et al. [69] added a criterion for case (i): they proposed using Tg as a criterion by considering polymers with Tg > 20 °C as solids.

They also considered the specific case of polymer gels, and suggested that, although it is not known whether they are benign for the environment, they should not be considered as particles, but as an independent polymer category whose possible environmental effects should be treated separately.

Some acrylic acid polymers can form hydrogels once in water. Rozman and Kalcikova [93] focused on the toxicity and biodegradability of some of these polymers used in cosmetic products. Three polymers in solid form (two cross-linked polyacrylic acid homopolymers, and a cross-linked polyacrylic acid copolymer) and one polymer in liquid form (a slightly cross-linked acrylate copolymer) were studied. Overall, the polymers had weak to moderate effects on aquatic organisms. The acrylate copolymer, however, had negative effects on oxygen consumption by nitrifying microorganisms at high concentrations, which could affect water treatment in wastewater treatment plants. A study at a more realistic concentration would be worthwhile. This polymer also significantly affected the bioluminescence of *Allivibrio fischeri* bacteria. Finally, all the polymers were not biodegradable in aqueous media according to the standard [94]. It has also been shown that the instillation of water-soluble cross-linked acrylic acid polymer into the trachea induces acute pulmonary inflammation, followed by fibrosis in rats [95].

Studies have also focused on the toxicity of non-ionic, anionic polyacrylamide to mussels [96] and cationic polyacrylamide to aquatic life [97]. These polymers are often used for wastewater treatment and also form gels when dissolved in water.

The Plastic Soup Foundation warned of the danger of “liquid microplastics” [98], whose long-term effects on health and the environment are poorly understood. The foundation defines these microplastics as liquid polymers with low biodegradability used in manufactured products, particularly cosmetics.

In 2024, several articles [99,100] focused on the detection of several polymers in wastewater corresponding to this definition, without qualifying them as “microplastics”.

According to REACH

The REACH regulation defines the “solid” state of polymers according to the GHS criteria, where a “solid” is anything that is not defined as a liquid or gas. The properties defining these different physical states are listed in Table 1.

For substances or mixtures whose Tf cannot be determined, they must be characterized according to the standard [101] standard method, or according to the fluidity test described in Appendix A of the European Agreement concerning the International Carriage of Dangerous Goods by Road (ADR) [102]. The first test involves measuring the flow of the mixture/substance through an inverted can at 38 ± 3 °C, and determines whether the sample is solid or liquid according to the result obtained. The second consists of determining the fluidity of the sample using a penetrometer carrying a constant load, whose penetration into the product to be tested is measured at a temperature of 35 ± 0.5 °C. If the penetration is insufficient, the product will be considered pasty rather than liquid.

#### 3.1.3. Chemical Structure

The word “plastic” is rarely defined in the articles we came across. Standard [74] defines a plastic as “a material which contains as its essential ingredient a high polymer (in practice larger than 10,000 Da) and which, at some stage of its transformation into a finished product, can be shaped by casting”. This definition includes thermoplastic and thermosetting polymers, but excludes elastomeric polymers. No mention is made of the polymer’s chemical structure (organic, inorganic, hybrid, etc.).

Based on the work of Hartmann et al. [69], several questions may arise: (i) should inorganic or hybrid polymers and (ii) elastomers be included as plastics?

According to the Literature

Elastomeric polymers

The abrasion of tires (made mainly of elastomers such as rubber or styrene–butadiene) would generate the release of 1,200,000 thousand billion particles into the ocean per year, or about 8.5% of the “plastics” found in the ocean, according to a 2020 report by The Pew Charitable Trusts [103].

Numerous studies [40,104,105,106] consider the microparticles released by this abrasion to be microplastics.

Tire particles, like other microplastics derived from thermoplastic or thermosetting polymers, are persistent and have harmful effects on the environment (release of additives and their transformation products with harmful effects [107], adsorption of heavy metals [108], and polymer toxicity [108]).

Inorganic/hybrid polymers

Similarly, silicone-based materials are not considered as plastics due to their elastomeric structure. They consist of a backbone of repeating—SiO_2_ groups with methyl or other functional groups grafted onto the silicon atom. Cross-linking of dimethylsiloxane leads to the formation of silicone elastomers, or “silicone rubber”. As such, these polymers are also “hybrids”, i.e., they possess both an organic and an inorganic structure.

Despite certain preconceived ideas, silicone rubber materials also contribute to plastic pollution and, consequently, to microplastics. Su et al., for example, have demonstrated the release of microplastics into the contained water after the wet-heating disinfection of feeding bottles with silicone rubber nipples [109]. Several studies have also demonstrated the presence of microplastics from silicone rubber in the environment [110,111].

Fang et al. [112] used Raman imaging to characterize microparticles and nanoparticles sampled from a silicone sealant (i.e., non-cross-linked) applied in a kitchen and on a roof and aged for several years. The samples were taken with a knife in an attempt to reproduce the cleaning of a joint. Their results open up the possibility that these sealants may release microplastics and nanoplastics.

Hartmann et al. [69] consider that this type of polymer should be regarded as a plastic. To date, however, little is known about the effects of silicone microplastics (whether cross-linked or not). Ekvall et al. [113] did not observe any acute toxicity of silicone nanoparticles from teats in *D. magna* after 72 h of exposure.

According to REACH

The REACH regulation considers elastomeric polymers to be plastics, as it prohibits the marketing of granules used to fill synthetic sports surfaces. These granules are used to increase the elasticity of synthetic sports turf, by filling the spaces between artificial grass strands, and are mainly made of ethylene–propylene–diene monomer rubber (EPDM) [114].

Regarding the chemical structure of the polymer, the REACH regulation excludes polymers containing no carbon atoms in their chemical structure from its definition. Hybrid polymers are therefore included as microplastics.

Finally, REACH [115] defines a polymer as a substance meeting the following two criteria:

- Be composed of more than 50% polymer molecules (i.e., molecules containing at least three monomer units covalently bonded to at least one other monomer unit or reagent).
- The mass percentage of molecules with the same molecular weight must not exceed 50%.

Accordingly, polymers containing more than 50% additives are not considered polymers under REACH. Nevertheless, it has been reported in the literature that several polymers such as polyamide or polyurethane can contain up to 50% by weight of additives [31].

#### 3.1.4. Origin

According to the Literature

Hartmann et al. [69] excluded natural polymers from the definition of plastic debris, since they are not plastics. However, in addition to synthetic polymers, they also include highly chemically modified natural polymers.

In another study, Zimmermann et al. [116] investigated the in vitro toxicity and chemical composition of bioplastics and/or biodegradable plastics and compared them with those of conventional plastics. Among the polymers tested were several natural polymers, cellulose and starch, in the form of finished products and granules, which were found to be the most toxic in vitro. However, the authors noted that the type of material does not predict toxicity, and that toxicity is highly dependent on the chemicals added to the plastic, such as monomers, oligomers, additives, lubricants, and unintentionally added substances. In addition, granulated products were less toxic than their finished counterparts. It was concluded that bioplastic and/or biodegradable materials had a toxicity similar to that of conventional plastics.

Therefore, although these polymers are of natural origin and for the most part chemically unmodified, the addition of other chemical substances in order to obtain the desired properties of use greatly increases their toxicity, and therefore makes them harmful to the environment.

Despite the wide variety of available additives, and hence of the chemical composition of plastics, these additives should also be taken into account when assessing the effect of MPs on the environment.

Furthermore, some chemically unmodified natural polymers are difficult to degrade, raising the question of their effect on the environment in the form of microparticles.

In the case of cellulose, Chen et al. [117] found microfibers in human lung tissues and tumors, the majority of which were composed of cellulose. The authors suggested that these microfibers could have effects similar to those of synthetic microfibers, in addition to possible indirect toxic effects due to the release of additives.

Liu et al. [118] drew attention to the abundance of natural (and semi-synthetic) microfibers in the environment, and the limited research conducted on them within the scientific community. However, they noted that many of the methods developed for microplastic study seem inadequate for the study of all microfibers created by human activity.

According to REACH

The REACH regulation excludes from the definition of “microplastic” any natural polymer, i.e., one whose polymerization mechanism occurs in nature, independently of its extraction process, and which is not a chemically modified substance.

According to Article I.II.3 of the REACH regulation, a “not chemically modified substance” is a “substance whose chemical structure remains unchanged even if it has been subjected to a chemical process or treatment or to a physical mineralogical transformation process, for example to remove impurities”.

#### 3.1.5. Persistence

1. Solubility in Water

According to the Literature

Hartmann et al. [69]. considered that polymers soluble to less than 1 mg/L in water at 20 °C should be included in the definition of microplastics, based on the REACH definition of a “poorly water-soluble” substance, used for long-term aquatic toxicity assessment. The REACH regulation had also considered this limit, but did not consider it appropriate to the problem of microplastics, which may be present in greater concentrations in the environment.

In the literature, the majority of authors consider water-soluble polymers as a problem in their own right, different from that of microplastics [93,119,120,121], as does the REACH regulation.

Although it seems logical that water-soluble polymers should be excluded from the definition of microplastics due to their different physico-chemical properties, they also contribute to environmental pollution, in concentrations that can exceed those of microplastics. It is therefore important to recognize the existence of this type of pollution, to study it, and to remedy it. Today, an increasing number of studies are being carried out on the impact and extent of this pollution, thanks to the development of suitable analytical methods [99,100,119,121].

According to REACH

In accordance with the provisions of the REACH regulation, the solubility of a polymer in water diminishes its “persistent” character. Consequently, water-soluble polymers are considered to be distinct from microplastics.

The REACH regulation excludes polymers that are soluble in water at more than 2 g/L at 20 °C and pH = 7. Accepted methods for assessing solubility are described in Table 2.

The 2 g/L limit was set in relation to derogation (b) of the definition of microplastic linked to biodegradability proposed by the REACH regulation. In fact, in ISO standards [122,123], which assess the ultimate aerobic biodegradability of plastic materials in aqueous media, the maximum solubility of samples to be assessed is set at 2 g/L. Thus, the REACH regulation considers that if microplastic particles are soluble in water at more than 2 g/L, they are sufficiently soluble for no particles to be present in the test system, and therefore biodegradation tests are not necessary.

Polymers in particulate form must be tested as they are placed on the market. Finally, it must be ensured that the particles are dissolved and do not form colloidal solutions, by checking for the presence of a Tyndall effect. If this is not the case, the test must be repeated with improved filtration.

2. Biodegradability

According to the Literature

Biodegradation is a set of contributions, some of biological origin and others not, acting synergistically to break down organic matter [124]. Biodegradation necessarily involves a bio-assimilation (or mineralization) stage. It may be accompanied by fragmentation and/or disintegration of the material. Disintegration of a plastic increases the surface area accessible to microorganisms, thus accelerating biodegradation if it is to take place. In particular, there are plastics that fragment without biodegrading: these are known as oxo-fragmentable plastics, or (incorrectly) oxo-biodegradable plastics.

According to the standard [125], fragmentation is “the set of concomitant and/or successive physical and/or chemical and/or biological phenomena leading to the disintegration of the material into increasingly smaller pieces. It can lead to partial or total separation of the material’s constituent(s) and to a greater or lesser loss of the material’s initial physico-chemical characteristics. Disintegration occurs when a material fractures into very small fragments (90% of grain size less than 2 mm—according to the standard [126])”.

The causes of fragmentation can be biological (physical, chemical, and especially enzymatic actions) or non-biological (photodegradation, thermodegradation, mechanical or chemical degradation).

A definition of degradation is also given in standard [125], February 2005, definition 3.2: “all concomitant and/or successive physical and/or chemical and/or biological phenomena leading, without exception, to molecular destructuration of all or part of the constituents. Degradation of a material is generally accompanied by fragmentation of the material preceding molecular destructuration” [124].

The OECD [127] characterizes biodegradation in six forms, from the most advanced to the least advanced:

- Ultimate: complete degradation of the compound into fully oxidized or reduced single molecules (such as CO_2_, CH_4_, NH_3_, and H_2_O)
- Primary (or biotransformation): modification of the chemical structure of a substance, resulting in the loss of a specific property.
- Ready: the product has passed certain selection tests specified for final biodegradability. These tests are so stringent that it is assumed that the compound will biodegrade rapidly and completely in an aerobic aquatic environment (in the presence of O_2_)
- Intrinsic: there is unequivocal evidence of biodegradation (primary or total) of the substance in any biodegradability test.
- According to its half-life (t_0.5_): the time required for 50% of the test substance to be transformed, when this transformation can be described according to a first-order kinetic law. This time is independent of the initial concentration.
- According to its disappearance time (DT_50_), i.e., the time required for its initial concentration to be divided by 2.

Concerning the biodegradable or non-biodegradable nature of microplastics, some authors do not consider this criterion in their definition, speaking of “biodegradable microplastics” [128,129,130,131,132,133,134,135,136,137,138]. Hartmann et al. [69] also do not take polymer biodegradability into account in their proposed definition of a microplastic.

Haider et al. [139] have demonstrated that biodegradation tests performed in artificial environments are not transposable to real-world conditions. Consequently, they stress the need for authentic, environmentally relevant field test conditions, as do Wang et al. [138].

Finally, several authors have highlighted the fact that biodegradable polymers can also be toxic to the environment due to the additives they contain [116,130,131].

According to REACH

The REACH regulation excludes from the definition of microplastics polymers that are biodegradable according to certain standards, divided into five groups, specified in Table 3.

The polymer is considered biodegradable according to the regulation if it meets the success criteria of groups 1 to 3. If the polymer does not meet these criteria, it must be tested according to group 4 or 5 criteria.

In the last case described, if the polymer is a product intended for horticultural or agricultural use, the success criteria will be different from those described in Table 3 and will vary according to their period of functionality. This period corresponds to “the period following product application during which the product performs its function”. In the case of seed coatings, this period is taken to correspond to the period of seed storage plus the period of seed growth prior to harvesting.

ECHA justifies this exclusion on the grounds that a biodegradable polymer “does not exhibit the same long-term persistence”, and therefore “does not contribute to the identified risk”.

#### 3.1.6. Conclusions

Microplastics are problematic because of the four following characteristics:

- Persistent in the environment;
- Small in size;
- Able to transport toxic elements;
- Rich in additives.

The REACH regulation is mainly concerned with persistence and size, but little with the toxicity of microplastics or their corresponding additives. It does not take into account manufactured nanoplastics such as those found in certain latexes, due to the difficulty of detecting and quantifying them at the time the regulations were drawn up. Elastomers and silicone polymers are also excluded, although they can also contribute to microplastic pollution. Finally, the natural origin of polymers does not guarantee their safety or biodegradability, as they are often used with additives. It would be preferable to take their formulation into account when assessing these parameters.

In the context of this review, and with the aim of highlighting the need for research into the substitution of microplastics in seed coatings, we define a microplastic according to the definition chosen by the REACH regulation, fully aware of the limitations of this definition.

### 3.2. Types of Microplastics

Microplastics can be divided into two categories according to their source: primary microplastics or secondary microplastics.

#### 3.2.1. Primary Microplastics

They are released directly into the environment in particle form by human action.

(i) They can be generated during the production phase of a manufactured product (e.g., intentionally added microplastics, such as polymer capsules used to encapsulate certain active ingredients, or polymer dispersions used as film-forming agents).
(ii) They can also be generated during the product’s use phase. The latter category includes, for example, tire particles resulting from road abrasion, or laundry water containing textile fibers.

Two conflicting opinions exist regarding the inclusion of cases in the definition of primary microplastic. Some studies [40,105,150] include case (ii) in the definition of primary microplastic, while others [151,152] do not.

#### 3.2.2. Secondary Microplastics

Secondary microplastics are defined as microplastics resulting from the fragmentation of larger plastic debris at different phases according to certain studies. These phases include the following:

(i) During use and maintenance (tires, textiles) and/or end of life (degradation and fragmentation of plastic waste in the environment).
(ii) Only at the end-of-life stage.

**Figure 5:**
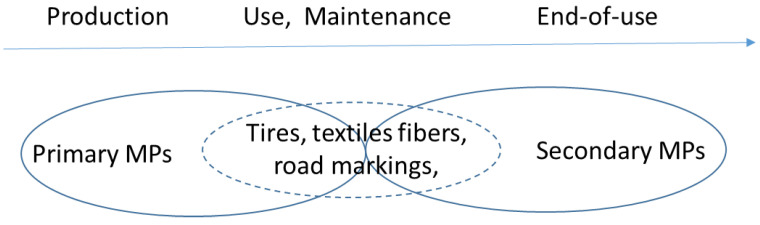
Diagram summarizing the various scenarios that may be employed to define primary and secondary microplastics.

Figure 5 provides a summary of the various scenarios observed.

**Figure 6:**
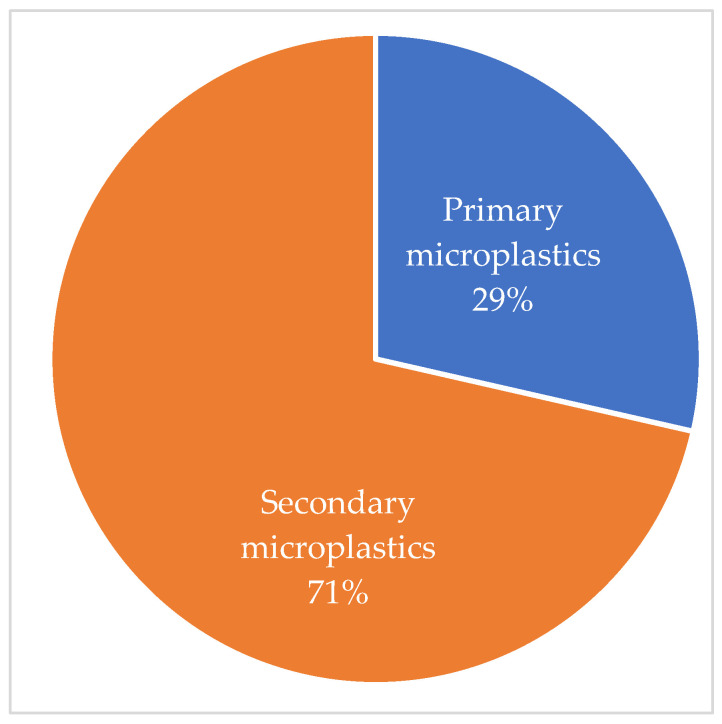
Distribution of each type of microplastic according to central estimates by Boucher and Friot [40].

The distribution of each type of microplastic is presented in Figure 6, based on the central estimates by Boucher and Friot [40], which consider only the share of cosmetics in manufactured microplastics. This distribution is made considering microplastics from tire abrasion and textile fiber washing as primary microplastics.

### 3.3. Most Common Microplastics

**Figure 7:**
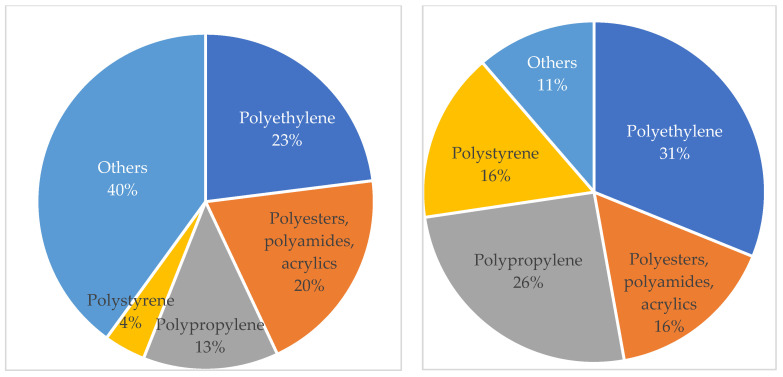
Comparison of the distribution of polymer types found in the marine environment obtained by Erni-Cassola et al. [5] (based on N = 40 studies) on the left, and the frequency of polymer identification in the marine environment obtained by Hidalgo-Ruiz et al. [139] (based on N = 42 studies) on the right.

Erni-Cassola et al. [5] conducted a meta-analysis of 40 studies on the distribution of plastic polymer types found in the marine environment. The analysis revealed the presence of 24 different polymer types, with polyethylene, the group of polyesters, polyamides, and acrylics (PP&A), polypropylene, and polystyrene being the most abundant. These findings align with the most widely produced plastics worldwide [3]. In 2012, Hidalgo-Ruz et al. [153] also listed the types of polymers encountered in the marine environment, not taking into account the number of particles counted, but the frequency with which they were identified in the literature. The results obtained are compared in Figure 7.

**Figure 8:**
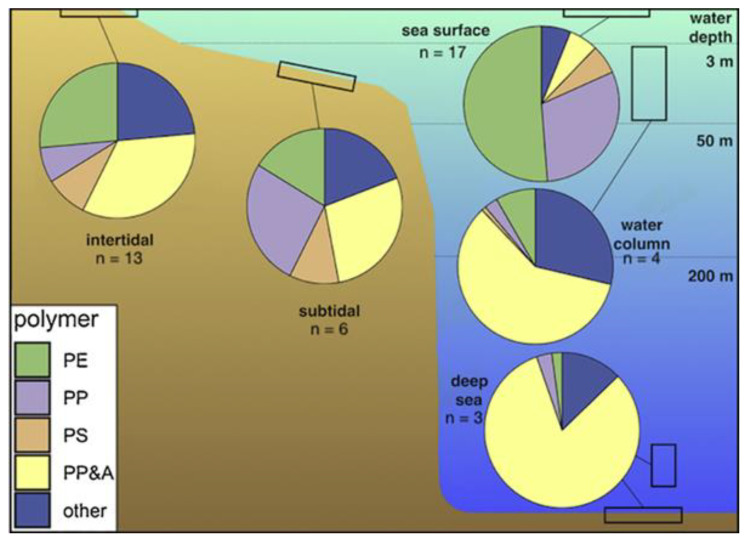
Prevalence of polymers encountered according to sampling medium from Cassola et al. [5].

A difference in the prevalence of polymer type was observed according to the type of environment: for example, PP&A polymers were more prevalent in deep sea (77%) and in water column samples (64%) than in surface water samples (5%), which may be attributed to their higher density than seawater [5]. The different types of polymers encountered depending on the sampling medium are illustrated in Figure 8.

A comprehensive analysis of the various sources of primary microplastics, encompassing both microplastics generated from the use and maintenance of products made of plastics (non-manufactured) and manufactured microplastics, is currently unavailable.

**Figure 9:**
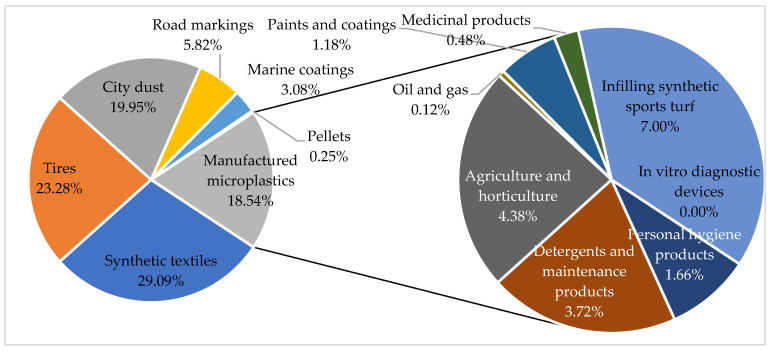
Assessment of the distribution of different sources of primary microplastics on a global scale: on the left, all primary microplastics (including non-manufactured MPs), and on the right, the share of manufactured MPs.

The data from Boucher and Friot [40] can be used to attempt to extrapolate the distribution of manufactured microplastic sources on a European scale to a global scale. This allows for the representation of the distribution of the different sources of manufactured and non-manufactured microplastics, as shown in Figure 9.

The data indicate that primary microplastics from treated seeds may represent 0.22% of all microplastics released into the environment. According to ECHA, this equates to 500 tons on a European scale.

We now turn our attention to the seed production process and the problems associated with the presence of microplastics.

## 4. Seeds

### 4.1. Seed Production Processes

Seeds are defined as a unit of plant reproduction, in contrast to grains, which are used for human or animal consumption [154]. Although seeds and grains are confused at harvest time, seeds undergo a much more laborious production process.

#### 4.1.1. General Production Diagram

Following harvesting, the seeds are subjected to a series of processes, including drying, cleaning, sorting according to size and density, and grading. This stage is also referred to as mechanical processing [155].

In certain instances, additional steps are incorporated into this process to improve the seed quality [156].

(i) Physiological preconditioning: This improves germination speed and capacity, resulting in faster, more uniform field emergence and a more uniform level of final establishment, particularly under unfavorable germination and growth conditions. A variety of techniques exist, as listed by Khan [157], which can be divided into two categories: those based on seed hydration (pre-soaking, wetting and drying, humidification, osmotic treatment, conditioning on a matrix, pre-germination), and those based on chemical or physical stimulation in addition to hydration (cold stratification, thermal shock, irradiation, oxygenation, hormonal treatment, salt treatment [158]).
(ii) Seed treatment: Crop protection products are applied to seeds for several purposes, including the control of seed-borne diseases and the protection of seeds and young plants from early attacks (diseases, parasites, pests) [159]. Biostimulants, fertilizers, and/or microorganisms can also be applied in addition to the treatment.
(iii) The addition of seed-coating agents to improve the distribution of the treatment on the seed, its adhesion, and any morphological changes.

The seeds are then quickly blow-dried at room temperature on a conveyor belt, after which they are bagged.

Steps (ii) and (iii) are separated here, but in practice, seed treatment and seed-coating agents can also be mixed and applied to seeds in a single step.

A more detailed examination of seed treatment and seed-coating agents is now in order.

#### 4.1.2. Seed Treatment

Seed treatment is defined as “the application of chemical and/or biological substances to seeds to prevent or control pathogens or pests” [160].

Seed treatment has two main functions: cure and prevention, i.e., cure the seed from any infection or infestation and prevent the seed and young plants from any early attack (disease, parasite, pest).

Depending on their function and nature, seed treatments can be divided into three categories [161]:

(i) Seed disinfection: This is a treatment against a pathogen that has infected the seed and is established in the seed coat or deeper parts. The seed is already infected by the pathogen.
(ii) Seed disinfestation: When the surface of the seed is contaminated with spores or other forms of pathogens, without being penetrated or infected, the seed is said to be infested with the pathogen. Chemical and fungicide dips, applied in powder or spray form, are effective disinfectants. Copper sulfate is a particularly effective disinfestant.
(iii) Seed protection: The seed is protected by coating it with crop protection products (fungicides, insecticides, bactericides) to prevent infection and damage by soil organisms, which it is particularly prone to during the early stages of its growth.

Seed treatments have been used by farmers for thousands of years to protect their crops. Early treatments consisted of solutions of lime and brine, used to prevent seed contamination by bacteria and fungi [160]. These solutions were supplanted by formulated products whose composition underwent evolution over time. Lime was supplanted in the early 1800s by copper sulfate, which proved more effective against common wheat decay. In 1920, copper sulfate was then supplanted by copper carbonate sprinkles, which were even more effective. Mercury-based products were also employed for their efficacy against several seed-borne biological problems. However, these products were banned in 1970 due to the risk of unintentional poisoning. In the 1980s, biological fungicides were introduced to the market, and developments in seed treatment methods led to the emergence of biological control in 1990.

E. Matyjaszczyk [162] demonstrated that the use of seed treatments for fungicides is often associated with a significantly lower release of active substances per hectare than foliar treatments. In 11 out of 13 cases, the difference was 8-fold or more.

In the present era, seed treatment chemicals may be classified into three categories: insecticides, fungicides, or bactericides [160]. Other products may be added during seed application, such as biostimulants and/or fertilizers, as well as a seed-coating agent, to improve treatment efficacy and certain technical and aesthetic properties of the seed. Collectively, these products form the “treatment slurry”.

#### 4.1.3. Seed Coating

Seed coating is defined as “the application of a material (in solid, liquid or dispersion form) to the surface of seeds, resulting in the formation of a continuous layer on that surface” [163].

This is an ancient practice, with the earliest documented evidence of its use dating back to 2000 years in China, where rice seeds were coated with mud to maintain their position in flooded fields. The first documented scientific reference to seed coating is a patent from 1866, which describes the use of wheat flour paste to treat cotton seeds with the aim of improving their germination rate.

The first seed coatings were commercially available in the 1930s, marketed by the British company Germains [164]. Their widespread use began in the 1960s, coinciding with the advent of precision seeding technology.

In 2020, a report by IHS Markit [165] estimated the value of the seed market to be 44.9 billion US dollars. The most widely sold seeds were corn (41% of the market), followed by soybeans (20%) and legumes (17%). According to the same report, the value of the market has risen sharply, with an increase of +45.6% between 2010 and 2020.

Seed coating is almost exclusively practiced on field crop and legume varieties, and to a lesser extent on grass, pasture, and flower seeds [166].

In practice, seed coating can be used for several reasons:

(i) When combined with seed treatment, it enables more precise and uniform application of the latter, as it provides greater adhesion to the seed [167,168].
(ii) When applied to the seed, it increases sowing precision, improves seed flow, and reduces the amount of dust released during seed handling [169,170,171].
(iii) Finally, it gives the seed a distinctive color, which enables it to be (a) differentiated from untreated seed, (b) easily identified, and (c) attractively presented. This also facilitates their visibility in the soil to check sowing quality [172].

There are three main types of seed coating, depending on the specific requirements of crops: film coating, encrustation, and pelleting. Other types of coating have also been reported, such as dry powder coating [173], agglomeration or conglomeration coating, or incorporation into extruded granules [174].

**Figure 10:**
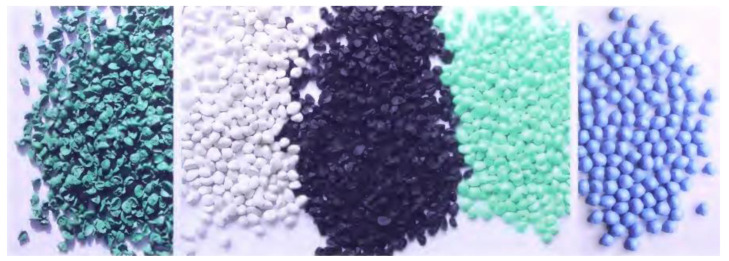
Uncoated (**center**), film-coated (**furthest left**), encrusted (**left**), and pelleted (2 different grades) onion seeds (**right**); photograph from The Encyclopedia of Seeds, Science Technology and Uses [175].

Film coating is the application of a thin layer of an external material (typically representing less than 10% of the seed’s weight). When the layer is thicker and represents 100 to 500% of the seed’sweight, the procedure is referred to as encrustation. This is carried out as long as the seed shape is still recognizable. When this is no longer the case, the procedure is called “pelleting” [166]. The three types of seed coating are illustrated in Figure 10.

Film coating is the process of applying a thin layer of material to the surface of the seed. Changes in mass, shape, and size are minimal, the mass typically being increased by a factor of less than 0.1 [175]. Therefore, it is employed for seeds that do not require greater bulk, weight, or uniformity in shape, such as rapeseed (*Brassica napus*), soybean (*Glycine max*), cotton (*Gossypium sp*), sunflower (*Helianthus annuus*), alfalfa (*Medicago sativa*), soft wheat (*Triticum aestivum*), or maize (*Zea mays*) [176].

This application technique has the advantage of being less ingredient-intensive and time-consuming than pelleting or encrustation [177].

The first patent relating to seed coating dates from 1964, by the German company VEB Leuna-Werke “Walter Ulbricht”. The process entails the application of “a plastic coating” to the surface of seeds, which renders them “resistant to cold and microorganisms” [178].

### 4.2. Seed Treatment Formulation

Seed treatment formulations are composed of numerous ingredients, which are frequently formulated to ensure stability, efficacy, and ease and safety of use. Each seed treatment is formulated with active ingredients adapted to each crop, target pests, and environmental conditions in which the seeds are planted [179]. Typical formulation ingredients are listed in Table 4.

Crop protection products frequently contain colorants and/or pigments which serve to enhance safety during handling, facilitate identification and assessment of distribution on the seed, and prevent spillage into the environment.

It is not uncommon for products belonging to the same category to be combined. This is particularly the case with corn seeds and fungicides. Similarly, plant protection products acting on different organisms can also be sold combined in a single product.

It is therefore essential that these ingredients are compatible with each other to avoid any problems prior to the application of the final seed treatment, such as a viscosity change, a chemical reaction, precipitation, and, in the event of a mixer stoppage, a phase shift.

Some crop protection products contain an adhesive polymer that acts as a seed film-coating agent in their formulation [215,216].

## 5. Seed Film-Coating Agents and Associated Functionalities

### 5.1. Functionalities of Seed Film-Coating Agents

Seed-coating agents are a separate category of product from fertilizers or crop protection products. They can be a simple adhesive polymer, or a more complex formulated product such as a seed film-coating agent, depending on the specific needs for which they are designed. In the case of seed pelleting, inert materials such as clay or talcum powder can also be applied.

For the purposes of this review, we are only interested in seed film-coating agents. To be effective, they must fulfill certain functions related to the seed performance factors mentioned in Section 4.1.3. For each of these functions, which are detailed below, we have included their associated physico-chemical properties.

#### 5.1.1. Adhesion to Seed

The primary function of the seed film-coating agent is to increase the adhesion of the seed treatment components to the seed surface. This function is typically performed by an adhesive polymer. An adhesive is defined as “a material that when applied to the surface of materials can join them together and resist separation” [217]. The adhesive polymer or seed film-forming agent, mixed with the other components of the seed treatment formulation, attaches them to the seed, potentially bonding them to the surface and “encapsulating” the seed.

Adhesion of the treatment to the seed is therefore the most important property to ensure its effectiveness. The adhesive polymer must (i) properly wet the seed surface in order to spread evenly and (ii) remain attached to the seed by chemical bonding, physical adsorption, electrostatic attraction, or mechanical locking [218].

**Figure 11:**
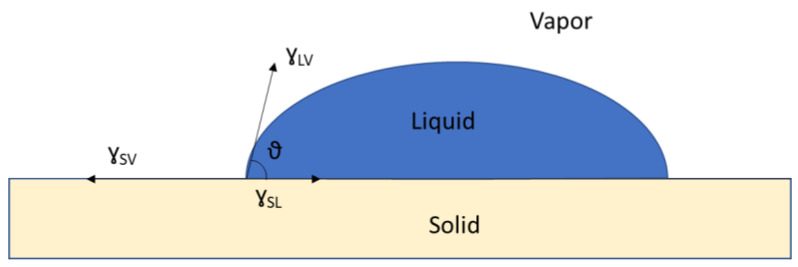
Representation of the solid, liquid, and vapor contact and corresponding components of the Young–Dupré equation.

The wettability of the seed surface depends on the surface tension of the crop protection products, the surface tension of the seeds, and their interfacial tension. Considering the seed as a solid with a perfectly smooth and atomically homogeneous surface, the contact angle ϑ between the seed treatment (liquid) and the seed (solid) is given by the Young–Dupré equation [219] (1), shown in Figure 11.

(1) $$\cos\left(\vartheta \right)=\frac{\sigma_{SV}-\sigma_{SL}}{\sigma_{LV}}$$

where *γ_SV_* is the surface tension of the solid (mN·m^−1^), *γ_LV_* is the liquid surface tension (mN·m^−1^), and *γ_SL_* is the interfacial tension between liquid and solid (mN·m^−1^).

Thus, for good wetting (ϑ < 90°), the surface tension or surface energy (J.m^−2^) of the solid must be greater than the interfacial tension or energy. High-energy surfaces are therefore easily wettable by many types of liquids. Conversely, high-surface-tension liquids are less likely to wet surfaces, due to their high affinity for themselves.

Most seed treatments consist mainly of water, which has a high surface tension but can be formulated to have a lower surface tension by adding surfactants. Pawlicki et al. [168] showed that the active ingredients tested (thiamethoxam, fludioxonil, metalaxyl-M and S-isomer, azoxystrobin, sedaxane) had poor adhesion to the surface of maize seeds. Several techniques are available to increase this adhesion.

Certain seed preconditioning processes, such as plasma treatment, reduce the surface energy by modifying the chemical composition of the seed [220,221,222,223].

The surface of the seed, called the spermoderm or testa, is more or less hydrophilic depending on its composition. The testa is a protective layer of dead cells covered by a cuticle in some species. The cuticle is a hydrophobic waxy layer that protects the seed and controls the permeability of its surface. The waxes that make up this layer are generally composed of a mixture of long unbranched saturated carbon chains, and an aliphatic polyester with long hydrocarbon chains called cutin [221].

Corn and sunflower seeds, for example, are coated with waxes composed mainly of long-chain esters (C42 to C58 for corn [224] and C40-C54 for sunflower [225], with C44, 46, and 48 predominating in both seeds). The cuticle makes the seed surface hydrophobic. For example, water forms a 120° contact angle on wheat [226] and a 90° angle on maize [227]. However, wax composition varies in quantity, uniformity, and coverage depending on the hybrid [228].

Zhong et al. [229] detected numerous hydrophilic hydroxyl groups on the surface of maize seeds by FTIR, which they attributed to the cellulose, hemicellulose, and lignin that make up maize, but they may have occurred due to the passage of the cuticle of the diamond during the analysis, which resulted in giving the composition of the lower layers. The authors also observed a reduction in seed abrasion when using a styrene–acrylic polymer with silane functional groups compared to a styrene–acrylic polymer alone. These groups could interact with the seed surface, improving the adhesion of the treatment, which could explain the observed reduction in abrasion. Polymers containing silane groups can form covalent C-O bonds with surfaces containing hydroxy groups [217].

It should be noted that no other article in the literature mentions a similar reasoning for developing a seed film-forming agent that adheres to the seed. Knowing the chemical composition of the seed surface, it may be interesting to design the seed film-forming formulation accordingly to maximize its adhesion.

Accinelli et al. [228] observed an increase in seed treatment adhesion after removing the cuticle from corn seeds. This technique is also described in an industrial patent [223].

Seed surface conditions can also influence treatment adhesion: for some fungicides, adhesion was greater on seeds with rough surfaces [230]. Plasma treatment also increases the seed surface roughness, which improves seed wettability [221,223].

Some sufficiently rough surfaces have been shown to be highly adherent to water despite their super-hydrophobicity [231]. Liquid in the form of a drop can remain attached to the solid despite the effect of gravity.

Most of the articles dealing with the adhesion of treatments to seed surfaces are old [230,232]. It can be assumed that it is mainly manufacturers who are interested in this problem, which they can solve by means of a suitable formulation and/or seed pre-treatment.

In industry, the CIPAC MT 194 test can be used to measure the percentage of active ingredients remaining on seeds after they are passed several times through a glass column and then dropped from a controlled height onto a sieve [233]. Measuring the contact angle also allows the assessment of physical adsorption adhesion.

Several articles suggest that the adhesion of the seed film-forming agent to the seed can be quantified by measuring the dust generated during its abrasion [228,234]. However, this dust may also be generated as a result of high friction between the seeds, and not only as a result of poor adhesion [229].

#### 5.1.2. Reducing Dust-Off

By increasing the adhesion of the components to the seed surface, the seed film-coating agent reduces the amount of dust generated when handling treated seeds.

In fact, the seed treatment can be abraded during (i) sowing, (ii) handling, and (iii) storage, resulting in pesticide-laden seed dust.

This dust, if fine enough, can be carried by the air to undesirable locations (adjacent fields, subsurface soil, aquatic environments). It can be lethal to bees and other pollinators (as in the case of neonicotinoids) [235] and pose health problems to farmers [163] due to their high concentration of active ingredients.

Aware of these issues, in 2011, Euroseeds, the non-profit association of the seed industry in the EU and EEA, in cooperation with the ESTA (European Seed Treatment Scheme), introduced a quality criterion related to the amount of dust released. In order to be marketed, seeds must not generate more dust than a threshold set by the ESTA. The thresholds for each type of seed are listed in Table 5.

These values are determined according to the Stauber–Heubach method, adapted for seeds. Initially, this is a European standardized test method normally used to determine dust generated during the handling of powders, granules, and tablets.

The test focuses on the quantification of airborne dust, as this is the most hazardous dust category (inhalation risks, environmental issues). The sample is placed in a stainless-steel drum with three baffles. Sample handling and transport are simulated by rotating the drum. A controlled airflow carries the released dust through a glass bottle, where it is aerodynamically sorted (heavier dust falls quickly) and sealed by a filter, where the airborne dust settles. The filter is then weighed, and the mass of dust is related to the number or mass of seeds tested.

The Stauber–Heubach method is recognized among seed manufacturers, and can be used to check whether the legal limit has been exceeded. However, when used to screen multiple seed film-coating agents, it may have certain limitations.

Indeed, given the low mass of seeds tested and the precision of the balance used (0.0001 g minimum), it becomes difficult to distinguish between several seed film-forming agents that generate little dust. This situation is becoming increasingly common as seed companies tend to set internal reference values, often much lower than the ESTA values. This limitation has also been noted in the literature [169,237] and, consequently, several other methods have been developed [228,229,237] using optical rather than mass measurements. However, unlike the Stauber–Heubach method, these methods are not standardized.

This limitation makes it difficult to rigorously demonstrate a relationship between certain physico-chemical properties of the seed film-forming agent and the measured dust and thus to better understand this phenomenon [169]. However, proof of this relationship in the scientific literature would allow seed film-coating agent manufacturers to carry out research and development work more easily and at lower cost, thanks to the implementation of faster and less equipment-intensive tests, such as certain tests used by paint manufacturers, as pointed out by Halecky et al. [169]. According to the same author, some companies have developed their own in-house dust measurement tests to differentiate products with low dust masses.

In addition to possible adhesion failure, this dust may also be due to high friction between the seeds, as mentioned above.

In his study, Herbert [234] developed a laboratory-scale abrasion test with conditions close to those of a Heubach test (rotation of treated seeds in a drum, and passage of an airflow at a controlled rate). The mass lost during the test is measured. Using data collected with this method for several latexes of different glass transition temperatures (Tg) containing the same polymer (ethyl vinyl acetate copolymer), Herbert observed that the lower the Tg of the latex, the lower the mass lost during the abrasion test. Thus, for the same polymer, latex with a lower Tg is preferred as an adhesive.

However, polymers with high glass transition temperatures are often associated with high strength, which can limit abrasion. Often, seed film-coating compositions combining polymers with low and high Tg can provide a satisfactory film, with high abrasion resistance and low tack [223].

Zhong et al. [229] observed a decrease in abrasion of treated seeds when polydimethylsiloxane was added in increasing amounts to a styrene acrylate polymer with silane groups applied to the seed as a seed film coating. The authors suggested that this result was due to a decrease in the coefficient of friction of the coating, caused by the addition of polydimethylsiloxane. It would be interesting to conduct further studies based on the tribology of seed film-coated surfaces.

#### 5.1.3. Improving Flowability

Seed rate per hectare and seed uniformity are the two main factors that determine crop yield. To control them, current seed drills are equipped with meters that provide real-time feedback on seed output.

The ability of the seed to flow freely, also known as “flowability”, is an essential property for good-quality seed. This property comes into play at every stage of seed transfer, from bagging to planting [238]. It is therefore directly related to plantability, which is the ability of the seed to flow through the seeder to provide uniform and constant spacing [239].

Flowability is influenced by both seed quality (irregular surface or wide particle size distribution) and seed film coating (poor drying, presence of tack). It is therefore important that the seed film coating provides the seed with a smooth surface and a high lubricity [169]. These characteristics can be achieved by adding seed film coatings.

There is no standardized seed flowability test, but according to Halecky et al. [169], most existing methods involve measuring the time it takes for a given number of seed to flow through a seeder. The shorter the time, the better the flowability. These authors demonstrated a relationship between the coefficient of friction between two surfaces of coated seed and the flowability: the lower the coefficient of friction, the better the flowability. They also demonstrated a relationship between flowability and the number of “singulations”, i.e., the number of times a seed is planted in a single location. Good flowability increases the number of singulations, which ensures good sowing.

It may also be interesting to look at the coefficient of friction between the coated seed and the seeder surface, although it can be assumed that its contribution to flowability is less than the coefficient of friction between two seeds.

Coatings are also applied to the surface of certain seed drills to improve seed flow and limit abrasion [240,241].

Other authors have taken an interest in seed flowability and measured it with a Revolution Powder Analyzer (Mercury Scientific Inc., Newton, CT, USA). The energy released during an avalanche or “burst energy” (kJ.kg^−1^) of a dry seed mass is measured in a rotating drum by imaging. The lower the energy, the better the flowability [170].

This technique can also be used to predict the flowability of cohesive powders. Trpelkova et al., for example, demonstrated the existence of a correlation between the fracture energy and the cohesion of the powders studied [242].

Amirkhani et al. [243] observed a negative effect of increasing the concentration of cross-linked potassium polyacrylate (PAL) on the surface of red clover seeds on their flowability. Above 8% PAL, flowability decreased with increasing PAL concentration. The authors suggest that increased PAL concentration may have led to greater absorption of ambient moisture by the polymers, resulting in reduced adhesion to the seeds and consequently reduced flowability.

Finally, Pawlicki et al. [168] attempted to correlate the flowability of several commercial seed film-coating agents with their chemical signatures, analyzed by time-of-flight secondary ion mass spectroscopy (ToF-SIMS) and their physical properties (adhesion to a glass substrate) obtained by atomic force microscopy (AFM). They compared three formulations with low (A), medium (B), and excellent (C) flowabilities, and observed that flowability improved as adhesion decreased, with A having the best adhesion, followed by B, and then C. They also observed local differences in adhesion to glass, which the authors suggested to be related to different chemical formulations, and probably to local differences in chemical composition. The authors concluded that the choice of adhesive polymer had the greatest influence on adhesion and consequently on flowability.

In patents, lipids and waxes are often added to the formulation to improve flow by reducing friction [168,223,244]. These waxes also improve seed appearance [244].

#### 5.1.4. Fast Drying

It is important to use a seed film coating that dries quickly on the surface of the seed. In fact, a seed film coating that is not completely dry, at least to the touch during bagging (about 5 min after treatment), will deposit on the walls of paper seed bags, the so-called “rub-off” [223]. The dose applied to the surface will therefore no longer be controlled, and the seed film coating will lose its effectiveness. Furthermore, it also contaminates surfaces. In the case of hand seeding, rub-off can cause health and safety problems [223].

Even after several months, the seed may not be completely dry [223]. In industry, the method [245] can be used to characterize the drying of seed film coatings [223,244]. This method characterizes the drying time of films under controlled conditions (temperature, humidity) according to several measures: (1) set-to-touch time, i.e., the time after which the film still sticks to the finger but no longer leaves a trace on it; (2) dust-free time: the time after which ambient dust no longer adheres to the film, measured when cotton fibers dropped from a certain height can be gently blown off the film; (3) tack-free time, determined using a suitable device (“Tack Tester”) or when an aluminum foil placed between the film and a 300 g weight can be removed without resistance at 90° after an application time of 5 s; (4) dry-to-touch time, when the film is no longer tacky to the touch and cannot be removed after light finger contact (no more “rub-off”); (5) dry-hard time: the time after which, by applying maximum pressure with the thumb, the mark left can be completely removed by polishing with a soft cloth; and (6) dry-through time, similar to the curing time, but this time by rotating the thumb while applying pressure. The film is considered dry-through when there is no peeling, delamination, or deformation of the film.

It can also be assumed that if the seed film-forming agent is not completely dry-through during handling or planting, this may reduce its adhesion to the seed, increasing abrasion and thus dusting. For example, a patent showed that the abrasion resistance of certain seed film-coating agents was better after three weeks of drying than immediately after application [223].

In addition, a seed film-coating agent that is not dry to the touch can cause flow problems such as seed clumping or seed bridging [223,244].

Finally, an extremely fast-drying seed film-coating agent will be an industrial advantage because it will result in faster seed production.

To the best of our knowledge, no scientific articles have been published on the drying speed of different seed film-coating agents, so the above information is taken from patents.

The drying method after application of the seed film-coating agent provided in the literature generally consists of drying at room temperature for 24 h [208,227,229] or longer [243,246], air blown [208,227] or not [229,243,246,247], at room temperature. Sometimes, the drying method is not mentioned [248,249]. Only one study mentions the use of a fluidized bed for seed drying [239].

#### 5.1.5. Improving Coverage

The application of a seed film-coating agent significantly improves the visual appearance of treated seeds, which must be completely and uniformly coated. Martins et al. [250] assessed the quality of coating by eye using a rating scale on 50 film-coated seeds. However, this type of visual measurement can lead to potentially inaccurate and unreliable results.

Another study examined the appearance of film-coated seeds using high-resolution photography, measuring color coordinates in the CIEL*a*b* space on 100 seeds [172] and calculating seed treatment coverage by summing the percentages of red-dominant and pink-dominant colors. The authors concluded that a seed treatment applied under industrial conditions provides significantly better coverage than a treatment applied in a mobile station.

In general, however, few scientific studies have focused on the visual appearance of film-coated seeds, as this is primarily an industrial criterion. Some information can be found in patents.

The seed film-coating agent must form a uniform film on the seed surface, which is facilitated by using a polymer with a minimum film-forming temperature (MFFT) lower than the application temperature. For example, the polymers commonly used in the industry have an MFFT and/or glass transition temperature below or slightly above 20 °C. If the polymer has a high glass transition temperature, the other ingredients in the seed film-coating agent can lower the MFFT by acting as plasticizers [223].

The same patent specifies the pigment volume concentration (PVC). This concentration is normally used in the paint industry and corresponds to the ratio between the volume of pigments and fillers and the volume of the dry film as a whole. Depending on its value, the film obtained will be more or less glossy. The critical pigment volume concentration (cPVC) is the pigment/filler concentration above which there is not enough polymer to encapsulate all of these particles. This concentration varies depending on the pigment/filler and the polymer. If the PVC is lower than the cPVC, the film consists mainly of polymer, making it cohesive and glossy. In the opposite case, the film is porous and dull because there are air pockets in the spaces between the particles instead of polymer [251]. In the patent, the PVC should preferably be 0.4 to 1 time the cPVC of the system under consideration, which corresponds to a rather glossy film.

#### 5.1.6. Ensuring Neutral or Good Effect on Seed Physiological Quality

##### Germination

Germination is an essential stage of plant growth. It is defined as the period between the onset of water uptake by the seed (imbibition) and the emergence of the embryonic axis (generally the radicle penetrating the tegument or pericarp). A seed whose radicle has emerged is therefore considered germinated, and the following events are associated with seedling growth and establishment. Germination capacity is defined as the percentage of seeds that germinate in a given period of time under conditions optimal for the seed [252]. Germination capacity is defined as the number of normal seedlings per 100 seeds. A seedling is considered normal if it correctly develops its essential parts (radicle and roots) [253]. Finally, the germination rate can be calculated in several ways: from the equation fitting the cumulative germination curve, or by considering the time required to reach a certain rate of germinated seeds (usually 50%) [254].

Seed film-coating agents can have a negative or positive effect on factors that affect germination:

(i) Water availability and uptake rate

Water uptake by the seed is critical to initiate germination. This is controlled by the difference in water potential (expressed in bars) between the seed and its external environment, e.g., soil water [175]. This stage can be influenced by the affinity of the seed film-coating agent for water and its thickness.

As early as 1959, an initial study by Dexter and Miyamoto [246] demonstrated that the application of hydrophilic colloids such as gelatin, agar, or sodium alginate to sugar beet seeds accelerated their water uptake in sand and their emergence from the soil under normal field conditions.

Application of a highly hydrophilic polymer (hydrolyzed starch grafted with polyacrylonitrile) to sweet corn seeds resulted in an increase in water uptake, respiration rate, and germination rate compared to untreated seeds at water potentials close to field capacity [247]. However, at lower water potentials (−1 to −1.5 MPa), the polymer has a negative effect on water uptake. At −1.0 MPa, the germination rate is slowed, and at −1.5 MPa, no germination is observed. The polymer also has a negative effect on respiration rate at these low water potentials. The authors propose two explanations for this phenomenon: (i) the respiration rate of a dry seed is low and increases only after water uptake; (ii) at low water potentials, water remains in the soil colloids, and water saturation of the polymer is not achieved. The polymer does not break due to this saturation, which can limit the movement of gases between the seed and its environment. Thus, according to the authors, “the polymer does not attract water from the surrounding soil, but immobilizes the water that comes in contact with it”. This type of polymer can therefore be used for non-irrigated crops in arid areas, where seeds are planted immediately before or after rainfall.

(ii) Presence of certain germination-inhibiting or germination-promoting molecules

The presence of a polymer film on the seed surface could prevent the leaching, oxidation, or deactivation of certain water-soluble germination-inhibiting molecules present in the pericarp, as demonstrated in sugar beet, and restrict the oxygen supply to the embryo, leading to a reduction in germination capacity in susceptible cultivars [255].

(iii) Presence of growth regulators

Seed film-coating agents also allow the controlled release of germination-promoting substances and growth regulators [202].

(iv) Influence on gas exchange

Sachs et al. [256] showed that coating bell pepper seeds with clay can cause a significant delay in the onset of germination and a reduction in germination rate compared to uncoated seeds. This effect is thought to be due to the physical properties of the coating that interfere with the diffusion of oxygen to the embryo, thereby limiting the availability of oxygen to the seed. However, the coating does not appear to have any effect on final germination capacity. It was also observed that bell pepper seeds were more affected than other vegetable seeds, which would suggest differences in oxygen affinity between different species during the germination process, and therefore different effects of the same coating on different species.

Finally, D. Scott [257] compared the effect of different adhesive polymers (gum arabic, methylcellulose, polyvinyl alcohol, acrylic polymer) on the germination of cocksfoot seeds treated with the same mixture (lime). All the treatments significantly improved the germination capacity, except the acrylic polymer, which slightly reduced it. The gum arabic treatment gave the best germination capacity.

##### Vigor

Vigor is as important to evaluate as germination, and it is complementary. According to ISTA, vigor is “the sum of seed characteristics that determine the level of activity and performance of the seed or seed lot for acceptable germination in a wide range of environments. It is not a simple measurable property, but a concept describing several characteristics related to the following aspects of seed lot performance:

(i) Rate and uniformity of seed germination and seedling growth
(ii) Seed emergence under adverse environmental conditions
(iii) Performance after storage, particularly maintenance of germination capacity.” [175]

Vigor assessment complements germination testing as it allows us to better predict the differences in emergence observed in the field between different seeds with high germination capacity and faculty, especially under harsh conditions.

In the literature, vigor is most commonly assessed by calculating vigor indices by multiplying germination capacity and seedling length (vigor index I) or average seedling dry mass (vigor index II) [258,259,260].

The authors Jacob et al. showed that the use of a hydrophilic polymer (Disco Clear, Incotec) preserved the quality of tomato seeds for a longer period (9 months instead of 3) than uncoated seeds, regardless of the packaging material and storage conditions. The authors suggest that the presence of the hydrophilic film acts as a physical barrier to the moisture vapor uptake and, consequently, coated seeds remain effectively protected from equilibrium with fluctuating relative humidity [261]. The vigor index obtained before storage was not significantly different between untreated seeds and seeds coated with the hydrophilic polymer studied. The authors conclude that this polymer is an inert material for the seed and has no direct physiological effect on it [261].

#### 5.1.7. Effective Active Molecule Release and Resistance to Leaching

Depending on their water solubility and hydrophilicity, the active ingredients in crop protection products can be highly leached by rainwater or irrigation water [107,262]. The release of these active ingredients is problematic because it leads to environmental pollution [263,264,265,266], especially since some active ingredients can be persistent in the environment, and to a significant loss of treatment efficacy [267].

The addition of a seed film-coating agent significantly reduces the leaching of seed treatments. This parameter can be assessed, for example, by measuring the resistance of the seed treatment to water after the application of the seed film-coating agent.

For example, Zhong et al. [229] immersed film-coated corn seeds in water for 24 h, observed the color of the water, and noted the water resistance of the treatment according to three ratings: good, fair, and poor. The authors observed a significant improvement in water resistance when applying the seed film-coating agents tested (synthetic polymers: acrylic, styrene–acrylic, styrene–acrylic with silane groups with and without polydimethylsiloxane). This test is also used by seed film-coating agent manufacturers [268].

However, the film formed by a seed film-coating agent must be sufficiently porous to allow the gradual release of active ingredients into the soil, such as systemic pesticides that provide a healthy growing environment for the seed. Polymers that swell on contact with water are often used for this purpose, with the swelling allowing the formation of pores [216].

To combine these two properties, hydrophilic polymers are often combined with hydrophobic polymers, for example by co-polymerization, which allows a more intimate and uniform blend than a physical blend of these two low-compatibility polymer types [216].

In summary, to perform their functions, seed film-coating agents must have a chemical composition that allows sufficient adhesion to the seed to limit the amount of dust generated, but not too high to ensure good flowability. They must dry quickly, forming a film as smooth as possible, and their mechanical properties must be such that the coefficient of friction between coated seeds is as low as possible (low Tg and/or high lubricity). Seed film-coating agents must also be sufficiently hydrophilic to release seed treatment active ingredients gradually upon contact with water, and to promote germination and seed vigor while being sufficiently hydrophobic (water resistant) to prevent rapid leaching.

With these criteria in mind, we can now turn our attention to formulating seed film-coating agents.

### 5.2. Seed Film-Coating Formulation

A seed film-coating agent may be the result of a complex formulation containing many ingredients. An overview of the functions of the ingredients used and the chemical family to which they belong can be obtained from patents (Table 6).

Thus, commonly used adhesive polymers contribute to microplastic pollution [269] both during production (unintentional release) and use (generation of microplastic dust [270]). It is therefore essential to find substitutes that are rapidly degradable in the environment and non-toxic while providing the same performance.

## 6. Seed Film-Coating Agent Substitution

The main challenge in replacing the polymers present in seed film-coating agents that are likely to generate microplastics is to maintain the same performance, and, in particular, their film-forming and adhesive properties, in order to meet the technical and functional requirements of the treated seeds. Indeed, synthetic polymers have the advantage of being easily modulated according to the desired properties by adding copolymers or functionalizing monomers, which makes them difficult to substitute.

Several adhesive polymers described in the literature can be considered as substitutes, since they may not be microplastics. The selected polymers are natural and/or water-soluble above 2 g/L at 20 °C and pH 7 and/or biodegradable according to specific OECD methods. Some polymers have been retained even though some biodegradability information is lacking, as they are likely to be biodegradable. However, biodegradability data from the literature should only be considered as initial screening data, as the biodegradability of polymers depends on the formulation in which they are used [271].

The use of REACH-compliant polymers in seed film-coating agents may present some drawbacks. Water-soluble polymers may also limit their ability to reduce leaching of active ingredients, thus potentially reducing seed treatment efficiency. It is necessary to use polymers with strong mechanical strength to ensure robust abrasion resistance, which is often lacking in natural polymers. Additionally, some natural polymers have high water absorption capacity, which can reduce adhesion to the seed [243]. While it was once possible to design synthetic polymers based on desired properties and performance, it is now necessary to intelligently combine biodegradable and/or natural polymers to tackle these challenges.

However, the use of natural and/or biodegradable polymers may have some more advantages over synthetic polymers. As said before, some polymers have a high water absorption capacity, which can lead to a beneficial growth-promoting effect [243]. It is possible that natural and/or biodegradable polymers act as a nutrient for soils when degraded.

Finally, REACH regulation does not take into account the potential toxicity of these polymers when degraded in the environment. An example is polyacrylamide, which can be used as a seed film-coating agent. Polyacrylamide itself is relatively non-toxic to humans, animals, poisons, and plants, but its monomer is neurotoxic, potentially carcinogenic, has high chronic and acute toxicity, and is harmful to aquatic life. Finally, its high solubility in water makes it highly mobile in the environment. During its use in soil, polyacrylamide is likely to be degraded, resulting in the release of its monomer into the environment [272]. These substitutes have been divided into four categories, i.e., polysaccharides, proteins, synthetic products, and other polymers, according to their origin and chemical structure, and are grouped in Table 7. The performance factors measured are also given.

Only one group of authors mentions the issue of microplastics and explicitly proposes a substitute [228,270]. The other substitutes were selected from other studies. The substitutes are divided into two categories according to their function: film-forming or absorbent. In fact, two approaches are often found when using adhesive polymers for seed film coating: a first approach where the aim is to limit the entry of water vapor into the seed, and to control the release of active ingredients (pesticides, biocontrol agents, growth-promoting agents) (film-forming function) and a second approach focused on the germination stage where, on the contrary, the aim is to provide a constant supply of water to the seed (absorbent function) to promote its growth under difficult conditions. Zvinavashe et al. [283] managed to combine these two approaches by designing a two-layer pelliculant. The first layer is a film-forming support for a growth-promoting bacterium, composed of hydrophobic silk fibroin with low oxygen permeability and trehalose, which serves as a carbon source for the bacterium. The second hydrophilic, super-absorbent layer holds water close to the seed. Finally, the majority of studies focus on the effect of seed treatment with the seed film-coating agent on seed physiological quality (measuring germination capacity, vigor, and yield), with only a few studies also looking at flowability or seed dust-off. Yet, these performance factors are very important, as they have a direct impact on the yield, efficacy, and safety of the seed treatment.

Incorporating new adhesive polymers in seed film-coating agents has the advantage of eliminating microplastics, making them more environmentally friendly. However, this also presents several challenges for the industry. Research and development of alternative polymers could entail additional time and costs. Existing alternatives are often of natural origin, which presents problems of sourcing and cost. A reformulation of the seed film-coating agent could often be necessary to obtain a stable and effective formula. In addition, the use of polymers with different rheological behaviors may require modifications to current seed film-coating processes. All of these constraints result in additional costs, which can be passed on to the price of treated seeds and have a direct impact on farmers. Finally, it is essential to study the fate and toxicity of new seed film-coating agents formulated without microplastics.

However, employing alternative polymers that are less persistent in soils and less toxic than those currently in use is crucial to protect the environment, maintain soil and organism health, and ensure food safety for the future.

## 7. Conclusions

This study provided information on the challenges and solutions for the substitution of microplastics in seed-coating films. The main challenges are multiple: the substitutes must offer the same level of performance as the microplastics by (i) increasing the adherence of the treatment to the seed, (ii) distributing the treatment more uniformly over the seed, (iii) reducing the amount of dust when handling treated seed, and (iv) improving seed flowability, which is particularly important during the sowing phase, all while maintaining seed physiological properties.

To date, there have been few studies examining microplastic substitution in seed film-coating agents, which are undeniably soil and air pollutants. However, this study was able to highlight the existence of potential substitutes in the literature, namely natural, water-soluble, or biodegradable polymers. It proposed several biopolymers to replace microplastics in the production of seed film-coating agents. Two major chemical families of biopolymers were identified in the literature: polysaccharides and proteins. Of these, 13 and 6 polymers, respectively, complied with REACH regulations. In addition, the majority of them demonstrated adhesion and dust reduction performances and maintained seed physiological quality.

Nevertheless, the natural origin of polymers does not guarantee their safety or biodegradability, which is not taken into account by the current REACH regulation. This could change in the future, along with other limitations such as the exclusion of certain polymers (silicone and elastomer). In addition, the use of natural and/or biodegradable polymers makes substitution more complex and can lead to additional challenges due to their different properties and therefore unsuitable performance. These challenges can be overcome by an intelligent combination of polymers in the formulation. In addition to these biopolymers, a list of regulatory-compliant synthetic polymers capable of providing the technological performance required for their use in seed coatings, as listed above, has also been found.

However, in most cases, these substitutes are not fully characterized from a physico-chemical or agronomic point of view (few flowability tests or Heubach tests, for example). In general, the relationship between the physico-chemical properties of the polymers used and their performance is poorly explained in the literature, and is probably known only by the manufacturers. Therefore, further research will be needed to determine which physical or chemical properties of alternative polymers should be evaluated to meet the functionalities required for seed coating.

The above research also shows that formulation adjustments are inevitable to satisfy the performance factors required for seed film-coating agents. One challenge associated with microplastic substitution is the ability to detect and quantify their presence in soil and air. This issue is not addressed in the present study, but it should be noted that to date, there are few published studies proposing solutions to the problem of detecting, identifying, and quantifying microplastics in complex matrices. These new challenges, which have been triggered by the necessity to make the transition to microplastic-free seed film-coating agents and which are driven by REACH regulations, provide an excellent opportunity to work on new polymer functionalities and thus lead to technological innovations and to acquire new knowledge of phenomena at the seed–polymer–environment interface.

## Figures and Tables

**Table 1:** Properties defining liquid, gaseous, and solid states according to the GHS.

Status Property	Liquid	Gaseous	Solid
Saturation vapor pressure at 50 °C	<300 kPa	>300 kPa	<300 kPa
Physical state at 20 °C and 101.3 kPa	Not completely gaseous	Completely gaseous	/
Melting temperature at 101.3 kPa	<20 °C	/	>20 °C

**Table 2:** ECHA-accepted methods for assessing water solubility.

		OECD LD 105	OECD LD 120
Low solubility (<10^−2^ g/L)	Sample loading	10 g/L	/
	Conditions	20 °C ± 0.5 °C, pH = 7, 24 h solubilization, N = 5 replicates	
	Solubilization method	Elution in water on a micro-column	
	Non-soluble part separation	Column filtration	
	Quantification method	GPC, HPLC, or dry matter content determination with gravimetric method	
High solubility (>10^−2^ g/L)	Sample loading	10 g/L	10 g/L
	Conditions	30 °C, then 20 ± 0.5 °C, pH = 7 24 h solubilization, then 24 h cooling N = 3 replicates	20 °C ± 0.5 °C, pH = 7 24 h solubilization N = 3 replicates
	Solubilization method	Flask method (heated stirring in closed flask then cooling)	Flask method (stirring in closed flask)
	Non-soluble part separation	Filtration or centrifugation	Filtration or centrifugation
	Quantification method	GPC, HPLC, or dry matter content determination with gravimetric method	GPC, HPLC, or dry matter content determination with gravimetric method

**Table 3:** Test methods for assessing polymer biodegradability according to REACH.

Name	Criteria (Biodegradability Rate and Duration)	Simulated Degradation Environment	Test Substance	Measurement Method
Group 1—Screening test methods and success criteria for demonstrating ready biodegradation				
OECD LD 301 B, C, D, F [127]	60% mineralization, 28 days	Aerobic aqueous environment	Organic substance soluble ≥ 100 mg/L in water, non-volatile, non-adsorbable	B: released CO_2_ C, D, F: biochemical oxygen demand (BOD)
OECD LD 310 [140]				Produced CO_2_
Group 2—Modified and improved screening test methods and success criteria for demonstrating ready biodegradation				
OECD LD 301 B, C, D, F [127]	60% mineralization, 28 days	Aerobic aqueous environment	Organic substance soluble ≥ 100 mg/L in water, non-volatile, non-adsorbable	B: CO_2_ released C, D, F: biochemical oxygen demand (BOD)
OECD LD 310 [140]				Produced CO_2_
OECD LD 306 [141]		Sea	Organic substance soluble ≥ 25–40 mg/L in water, non-volatile, non-adsorbable on glass	Dissolved organic carbon (DOC)
Group 3—Screening test method and success criteria for demonstrating intrinsic degradation				
OECD LD 302 C [142]	70% mineralization, 14 days	Aerobic aqueous environment	Organic substance dispersible/soluble in the medium	BOD
Group 4—Screening test methods and success criteria for demonstrating degradation against a reference material				
EN ISO 14852:2021 [122]	90% degradation compared with reference material, 6 months	Natural aquatic environment	Natural and/or synthetic polymers, copolymers, or mixtures thereof. Plastic materials containing additives Water-soluble polymers	Released CO _2_
EN ISO 14851:2019 [123]				
EN ISO 19679:2020 [143]	90% degradation compared with reference material, 24 months	Sediment/sea interfaces	Non-floating plastic materials	
EN ISO 18830:2016 [144]			Plastic materials	BOD
ISO 22404:2019 [145]		Marine sediments	Non-floating plastic materials	
EN ISO 17556:2019 [146]		Soil	Natural and/or synthetic polymers, copolymers or mixtures thereof. Plastic materials containing additives Water-soluble polymers	Released CO_2_
Group 5—Simulation test methods and success criteria for demonstrating degradation under relevant environmental conditions				
OECD LD 307 [147]	Degradation half-life < 180 days	Soil	Non-volatile organic substance	Half-life (monitoring of the test substance concentration in the medium)
OECD LD 308 [148]		Aquatic sediments		
OECD LD 309 [149]	Degradation half-life < 60 days	Surface water		

**Table 4:** Typical constituents of the slurry treatment.

Constitute	Active Molecule	Chemical Family	References
Water	/	/	/
Insecticide	Tefluthrin	Pyrethroid	Ferracini et al. [180]; Agatz and Brown [181].
	Thiamethoxam Clothianidin	Neonicotinoid	Agatz and Brown [181]; Suganthi et al. [182]. Agatz and Brown [181]; Klatt et al. [183].
Nematicide	Abamectin	Avermectin	Clifton et al. [184].
	*Pasteuria Nishizawae*	Bacteria	
Fungicide	Metalaxyl Metalaxyl-M	Acylamine	Chaudhari et al. [185]; Sharma and Madhavan [186].
	Fludioxonil	Phenylpyrrole	Alvarez et al. [187]; Panozzo et al. [188].
	Sedaxane Penflufen	Pyrazole-carboxamides	Panozzo et al. [188]; Zhang et al. [189]. Panozzo et al. [188]; Jayaweera and Ray [190].
	Prothioconazole Triticonazole Difenoconazole Tebuconazole	Triazole	Sjarpe et al. [191]; Hakki et al. [192]. Sjarpe et al. [191]; Ye et al. [193]. Mongiano et al. [194]; Hysing and Wiik [195]. Ye et al. [193]; Nampeera et al. [196].
	Trifloxystrobin Pyraclostrobin	Strobilurin	Ye et al. [193]; Gireesha et al. [197]. Hakki et al. [192]; Choudhary et al. [198].
	Captan	Phthalimide	Hakki et al. [192]; Kurilova and Bushneva [199].
	Abamectin	Avermectin	Kumar et al. [200]; Bagri et al. [201].
Corvid repellent	Ziram		Kumar et al. [200].
Microorganisms	Growth-promoting bacteria	*Bacillus* *Pseudomonas* *Azospirillum* *Azotobacter*	Sjarpe et al. [191]; Camacho et al. [179]. Paravar et al. [202].
	Fungi	Rhizobia Trichoderma	Rocha et al. [203].
Fertilizers	Zinc		Rasmussen and Boawn [204].
	Nitrates Molybdenum Cobalt		Lana et al. [205].
Additives	Petroleum-based oils Natural or modified vegetable oils Surfactants Rheological agents		Chester L. Foy [206].
Seed-coating agents	Adhesive polymers	Methylcellulose Carboxymethylcellulose Gum arabic	Hathcock and Dernoeden [207]. Abdelzaher et al. [208]. Zhang et al. [209].
	Adhesive formulations		
Biostimulants	Microorganisms		Capo et al. [210].
	Algae extracts		Supraja et al. [211].
	Lignocellulosic extracts		Mutlu-Durak and Kutman [212]; Campobenedetto et al. [213].
	Trace elements		Campobenedetto et al. [213].
	Plant hormones		Pereira et al. [214].

**Table 5:** Dust threshold for each seed according to ESTA [236].

Seed	Threshold Value
Maize	0.75 g/100,000 seeds
Rapeseed	0.50 g/100,000 seeds
Sugar beet	0.25 g/100,000 seeds
Sunflower	0.40 g/100,000 seeds
Cereals	4 g/100,000 seeds
Carrots, endives	0.1 g/100,000 seeds
Onions	0.2 g/100,000 seeds
Sweet corn	0.75 g/100,000 seeds
Green and seed beans	0.4 g/100,000 seeds
Fodder peas	0.2 g/100,000 seeds
Cotton	6 g/100,000 seeds

**Table 6:** Functions of ingredients commonly used in seed film-forming formulations, and associated chemical families.

Function	Examples	References
Water	/	/
Adhesive polymer(s)	Polyvinyl acetate dispersion, styrene acrylate copolymer dispersion, ethylene acrylic copolymer dispersion	US 2018/0325104 A1 [244] AU 2015276335 B2 [223]
Flow agent(s)	Wax-based compounds: carnauba wax, paraffin wax, polyethylene wax, beeswax, polypropylene wax	US 2018/0325104 A1 [244] AU 2015276335 B2 [223]
Surfactant(s)	Non-ionics: Sugar alcohols, fatty alcohols, alkylphenols, or ethoxylated, propoxylated glucose, PEG, PPG Glycerol esters, sorbitan, pentaerythritol, sorbitol, sucrose Alkylamines, ethoxylated fatty amines, alkanolamides Anionics: Sulfates, phosphate, PEG sulfonate, PPG Carboxylic acids and copolymers of carboxylic acids, sulfates, sulfonic acids, phosphates (lignin sulfonate, alkylaryl sulfonates) Cationics: Amino acid derivatives Alkylammonium salts Amphoterics: Betaine-type amides	US 2018/0325104 A1 [244]
Pigment paste/pigments	Monoazo organic pigments (Pigment Red 112, 2, 48:2, Yellow 74, Orange 5), phthalocyanines (Pigment Blue 15:3, Green 36), oxazine (Violet 23), carbon (Black 7), TiO_2_ (White 6)	US 2018/0325104 A1 [244]
Dye(s)	Anthraquinone, triphenylmethane, phthalocyanine and derivatives, and diazonium salts	AU 2015276335 B2 [223]
Opacity agent(s)	CaCO_3_	AU 2015276335 B2 [223]
Anti-foaming agent(s)	PEG, glycerol, mineral oils, silicone-based oils (PDMS), polyethers, polyacrylates	US 2018/0325104 A1 [244]
Rheological agent(s)/stabilizer(s)	Agar, carboxymethyl cellulose, carrageenan, chitin, fucoidan, ghatti gum, gum arabic, karaya gum, laminarin, locust bean gum, pectin, alginate, guar gum, xanthan gum, diutan gum, and gum tragacanth, bentonite clays, HEUR thickeners (hydrophobically modified ethoxylated urethane), HASE thickeners (hydrophobically modified alkaline swellable emulsion) and polyacrylates.	US 2018/0325104 A1 [244]
Antifreeze agent(s)	Ethylene glycol, propylene glycol	US 2018/0325104 A1 [244]
Preservative	Biocides: MIT (2-methyl-4-isothiazolin-3-one), BIT (1,2-benzisothiazolin-3-one), CIT (5-Chloro-2-methyl- 4-isothiazolin-3-one), Bronopol	US 2018/0325104 A1 [244]
pH regulator	Citric acid, NaOH	

**Table 7:** Adhesive molecules cited in the literature for use as seed film-coating agents.

Polymer(s)	Function	Natural (N)/Soluble in Water (S)/Biodegradable (B)	References	Performance Factor Measured
Polysaccharides				
Methylcellulose	Film-forming	Not N, **S**, ND	Samal et al. [273]; Rocha et al. [203]; Javed et al. [177];	Physiologic quality of seeds
Ethylcellulose		Not N, **S**, ND	Samal et al. [273]	
Carboxymethylcellulose		Not N, **S**, not B according to OECD 301E [274]	Rocha et al. [203] Abdelzaher et al. [208]	
Hydroxypropyl cellulose		Not N, **S**, not B according to OECD 301D [275]	Samal et al. [273]	
Sodium alginate + Ca(NO_3)2_		**N, S, B** according to OECD TG 301F [276]	de Castro et al. [277]	
Chitosan		Not N, not S, **B** according to OECD 301B [278]	Rocha et al. [203]	
Chitosan lignosulfonate		Not N, not S, ND	Thobunluepop [279]	
Gum arabic		**N, S,** ND	Zhang et al. [209]; Javed et al. [177]; Rocha et al. [203]	
Pre-gelatinized starch		**N, S, B** according to OECD TG 301B [280]	Accinelli et al. [227]	Seed dust-off and physiologic quality of seeds
Pulullan		**N, S,** ND	AU 2015276335 B2 [223]	Drying speed, seed dust-off, flow ability, physiologic quality of seeds
Guar gum	Absorbent	**N, S, B** according to OECD TG 301 F [281]	Chen et al. [282]	Seed dust-off, flow ability, physiologic quality of seeds
Hydroxypropylguar		Not N, **S**, ND		
Denatured starch + chitin + glycerol (0.2% *w*/*w*)	Film-forming	**N**, **S**, ND	Accinelli et al. [249]	Seed dust-off; physiological quality of seeds
Proteins				
Soy flour	Film-forming	**N**, not S, ND	Javed et al. [177]	Physiologic quality of seeds
Zein		**N**, not S, ND	AU 2015276335 B2 [223]	Drying speed, seed dust-off, flow ability, physiologic quality of seeds
Casein		**N**, not S, ND		
Gelatin		**N**, not S, ND		
Soy protein isolate + Starch + arabic gum		**N**, not S, ND	Accinelli et al. [228]	Seed dust-off, physiological quality of seeds
Silk fibroin/trehalose inner layer + pectin/caboxymethylcellulose outer layer	Film-forming + absorbent	**N, not S, ND**	Zvinavashe et al. [283]	Physiological quality of seeds
Synthetic polymers				
Polyvinylpyrrolidone	Film-forming	Not N, **S**, not B according to OECD TG 302B [284]	Halecky et al. [169]; Samal et al. [273]; Kimmelshue et al. [285]	Seed dust-off, physiological quality of seeds
Polyvinyl alcohol		Not N, **S**, **B** according to OECD TG 302B [286]	Javed et al. [177]; Halecky et al. [169]	Seed dust-off, physiological quality of seeds
Polyacrylamide (PAM)	Absorbent	Not N, **S**, ND	Amirkhani et al. [243]; Su et al. [287]	Flow ability, physiological quality of seeds
PAM with graphite (PAM + G)		Not N, **S**, ND	Amirkhani et al. [243]	
Acrylamide and sodium acrylate copolymer		Not N, **S**, ND	Su et al. [287]	Physiological quality of seeds
Acrylamide and potassium acrylate copolymer		Not N, **S**, not B according to OCDE TG 302B [288]	Su et al. [287]	
Sodium polyacrylate		Not N, **S**, ND	Su et al. [287]	
Others				
Lignin/polyethylene glycol copolymer	Film-forming	Not N, not S, ND	Gu et al. [216]	Release of active ingredients
Shellac		**N**, not S, ND	AU 2015276335 B2 [223]	Drying speed, seed dust-off, flow ability, physiologic quality of seeds

## Data Availability

Not applicable.
